# Protective immune trajectories in early viral containment of non-pneumonic SARS-CoV-2 infection

**DOI:** 10.1038/s41467-022-28508-0

**Published:** 2022-02-23

**Authors:** Kami Pekayvaz, Alexander Leunig, Rainer Kaiser, Markus Joppich, Sophia Brambs, Aleksandar Janjic, Oliver Popp, Daniel Nixdorf, Valeria Fumagalli, Nora Schmidt, Vivien Polewka, Afra Anjum, Viktoria Knottenberg, Luke Eivers, Lucas E. Wange, Christoph Gold, Marieluise Kirchner, Maximilian Muenchhoff, Johannes C. Hellmuth, Clemens Scherer, Raquel Rubio-Acero, Tabea Eser, Flora Deák, Kerstin Puchinger, Niklas Kuhl, Andreas Linder, Kathrin Saar, Lukas Tomas, Christian Schulz, Andreas Wieser, Wolfgang Enard, Inge Kroidl, Christof Geldmacher, Michael von Bergwelt-Baildon, Oliver T. Keppler, Mathias Munschauer, Matteo Iannacone, Ralf Zimmer, Philipp Mertins, Norbert Hubner, Michael Hoelscher, Steffen Massberg, Konstantin Stark, Leo Nicolai

**Affiliations:** 1 Department of Medicine I, University Hospital, LMU Munich, Munich, Germany; 2 DZHK (German Centre for Cardiovascular Research), partner site Munich Heart Alliance, Munich, Germany; 3 Department of Informatics, Ludwig-Maximilian Universität München, Munich, Germany; 4 Anthropology and Human Genomics, Faculty of Biology, Ludwig-Maximilians University Munich, Munich, Germany; 5 Max Delbrück Center for Molecular Medicine (MDC) and Berlin Institute of Health (BIH), Berlin, Germany; 6 Laboratory for Translational Cancer Immunology, Gene Center, LMU Munich, Munich, Germany; 7 Department of Medicine III, University Hospital, LMU Munich, Munich, Germany; 8 Division of Immunology, Transplantation and Infectious Diseases, IRCCS San Raffaele Scientific Institute, 20132 Milan, Italy; 9 Vita-Salute San Raffaele University, 20132 Milan, Italy; 10 Helmholtz Institute for RNA-based Infection Research, Helmholtz-Center for Infection Research, Würzburg, Germany; 11 Max von Pettenkofer Institute & GeneCenter, Virology, Faculty of Medicine, LMU München, Munich, Germany; 12 German Center for Infection Research (DZIF), Partner Site Munich, Munich, Germany; 13 COVID-19 Registry of the LMU Munich (CORKUM), University Hospital, LMU Munich, Munich, Germany; 14 Division of Infectious Diseases and Tropical Medicine, University Hospital Ludwig-Maximilian University Munich, Munich, Germany; 15 Gene Center and Department of Biochemistry, University Hospital Ludwig-Maximilian University Munich, Munich, Germany; 16 Department of Medicine II, University Hospital, LMU Munich, Munich, Germany; 17 DZHK (German Centre for Cardiovascular Research), Partner Site Berlin, 13347 Berlin, Germany; 18 Experimental Imaging Centre, IRCCS San Raffaele Scientific Institute, 20132 Milan, Italy

**Keywords:** Viral infection, Inflammatory diseases, SARS-CoV-2, Infection

## Abstract

The antiviral immune response to SARS-CoV-2 infection can limit viral spread and prevent development of pneumonic COVID-19. However, the protective immunological response associated with successful viral containment in the upper airways remains unclear. Here, we combine a multi-omics approach with longitudinal sampling to reveal temporally resolved protective immune signatures in non-pneumonic and ambulatory SARS-CoV-2 infected patients and associate specific immune trajectories with upper airway viral containment. We see a distinct systemic rather than local immune state associated with viral containment, characterized by interferon stimulated gene (ISG) upregulation across circulating immune cell subsets in non-pneumonic SARS-CoV2 infection. We report reduced cytotoxic potential of Natural Killer (NK) and T cells, and an immune-modulatory monocyte phenotype associated with protective immunity in COVID-19. Together, we show protective immune trajectories in SARS-CoV2 infection, which have important implications for patient prognosis and the development of immunomodulatory therapies.

## Abstract (web summary)

Infection with SARS-COV-2 can result in self-limited upper airway infection or progress to a more systemic inflammatory condition including pneumonic COVID-19. Here the authors utilise a multi-omics approach to interrogate the immune response of patients with self-limiting upper respiratory SARS-CoV-2 infection and reveal a temporal immune trajectory they associate with viral containment and restriction from pneumonic progressive disease.

## Introduction

Due to its high contagiousness and an approximate case fatality rate of 1.0–2.3% the COVID-19 pandemic has confronted the world with a major health care and economic challenge^1–3^. Approximately 10% of cases have a severe disease course^4^. Extensive data now underlines immunopathology, the concept of self-inflicted damage to tissues by the immune system, as an important factor contributing to disease progression in COVID-19^4–8^.

Various detrimental pathways have been identified to drive immunopathology in COVID-19: A cytokine storm with high levels of IL-6 and some similarity to chimeric antigen receptor (CAR) -T cell hyperinflammation was postulated^9,10^. Recent data highlighted a failure in host interferon I and III responses associated with progression to severe COVID-19^11–13^. In contrast to influenza, severe COVID-19 has also been associated with impaired Interferon-y expression in T cells^4,7,14^. Seminal work revealed auto-antibodies against interferon pathway related proteins and inherent dysfunctional mutations in a significant proportion of severe COVID-19 cases^15,16^. T cells in severe cases show upregulation of exhaustion and apoptosis markers^5^. Apart from adaptive immunity, the concept of a dysregulated innate immune cell axis evolved consistently across studies, with an expansion of HLA-DR^low^ monocytes and a surge in (premature) neutrophils, which in turn cause vascular inflammation and immunothrombosis in the lung and remote organs^17–19^.

Interestingly, most infected patients, even those with known risk factors like advanced age, hypertension, or obesity, efficiently clear SARS-CoV-2 without developing pneumonia^20,21^. This highlights the ability of the host immune system to mount an effective response and contain the virus in the upper airways without causing pulmonary damage in the majority of cases^22^.

Yet, the pathways that provide protective immunity in SARS-CoV-2 infection are less well understood. Outpatients and patients with oligo- and asymptomatic SARS-CoV-2 infection have been underrepresented in mechanistic studies.

To better understand how protective immune response and immunopathology differ in SARS-CoV-2 infection, we launched a multi-cohort multi-omics study characterizing the immune response in ambulatory and non-pneumonic infected individuals. With this experimental approach, we first used a well characterized, exploratory cohort of high-risk patients for hypothesis generation and subsequently confirmed our findings in a large cohort of outpatient SARS-CoV-2 infected individuals. Our approach allowed us to identify protective cell responses in these individuals by contrasting them with COVID-19 pneumonia cases and healthy controls in a longitudinal manner.

We identify a distinct immunological signature of successful viral containment, featuring a prominent, early interferon stimulated gene (ISG) upregulation across circulating immune cell subsets. This systemic ISG signature does not correlate with plasma interferon levels or with mucosal antiviral immune responses early in disease. In addition, reduced cytotoxic potential of Natural Killer (NK) and T cells compared to pneumonic patients and control subjects, as well as a monocyte phenotype with immune-modulatory properties are hallmarks of protective immunity.

## Results

### Longitudinal clinical and cellular characteristics of the successful and failing immune response to SARS-CoV-2

**Fig. 1:**
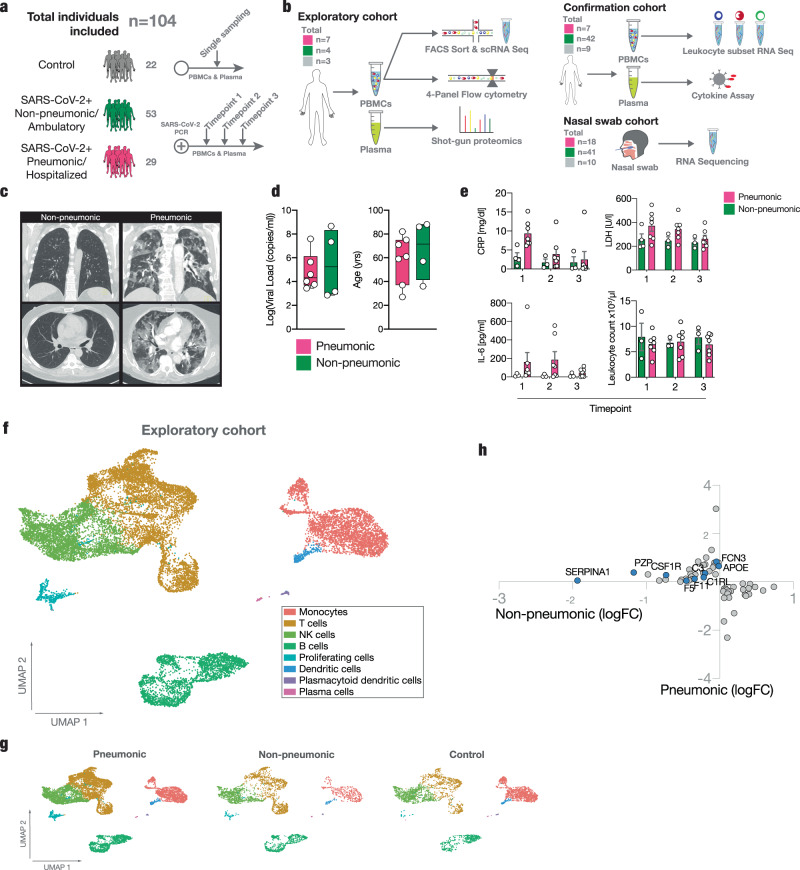
Study overview and longitudinal clinical and cellular dynamics in non-pneumonic and pneumonic SARS-CoV-2 infection. **a**, **b** Experimental setup and processing pipeline. **a** Plasma and PBMCs of *n* = 22 non-infected Controls, *n* = 29 pneumonic/hospitalized COVID-19, *n* = 53 non-pneumonic/ambulatory infected patients were sampled **b** Exploratory cohort: longitudinal samples from 11 pneumonic and non-pneumonic infected patients and one timepoint from three non-infected control patients were used for shotgun plasma proteomics, 50-dimensional flow cytometry or single-cell RNA sequencing. scRNA sequencing: *n* = 12 patients (*n* = 6 pneumonic, *n* = 3 non-pneumonic, *n* = 3 control), 4-panel flow cytometry: *n* = 11 patients (*n* = 7 pneumonic, *n* = 4 non-pneumonic), shot-gun proteomics: *n* = 14 patients (*n* = 7 pneumonic, *n* = 4 non-pneumonic, *n* = 3 control). Not all patients from one cohort were included into every analysis due to a lack of respective sample availability. Confirmation cohort: Longitudinal samples were used for leukocyte subset RNA sequencing: *n* = 55 patients (*n* = 39 ambulatory patients, *n* = 7 hospitalized patients, *n* = 9 controls) and cytokine assays *n* = 56 (*n* = 40 ambulatory patients *n* = 7 hospitalized patients, *n* = 9 controls). Nasal swab cohort: RNAseq of nasal swabs *n* = 69 (*n* = 41 ambulatory patients *n* = 18 hospitalized patients, *n* = 10 controls). Nasal swabs were included from both hospitalized and ambulatory patients that were either already included in the two independent cohorts mentioned above (*n* = 37) or were additionally recruited (*n* = 32). **c** Representative axial and coronal computed-tomographic scans of hospitalized pneumonic and non-pneumonic infected patients. **d** Baseline characteristics of the exploratory group: log_10_ of viral load as measured in copies/ml of upper respiratory tract swap samples. *n* = 6 pneumonic COVID-19, *n* = 4 non-pneumonic infected patients. Age in years of patients. *n* = 7 pneumonic COVID-19, *n* = 4 non-pneumonic. Box-and-whiskers plot (median, IQR and min-max). **e** Qualitative longitudinal clinical laboratory values of CRP (mg/dl), LDH(U/l), IL-6 (pg/ml), and total leukocyte count (1000/µl) at time points 1–3. *n* = 7 pneumonic COVID-19 per time point, *n* = 3 non-pneumonic COVID-19 patients per time point, except TP1 CRP and LDH *n* = 4. **f** Integrated UMAP representation of the pooled exploratory cohort showing the assigned cell populations. **g** Integrated UMAP representation of the sequenced samples showing the assigned cell populations per group for all time points. **h** Plot depicting significantly (*p* < 0.05) differentially expressed proteins in plasma samples pooled across all three time points. Log fold changes are computed relative to the control proteins’ expression. All error bars are mean ± s.e.m. unless otherwise noted. Source data are provided as a Source Data file.

We compared high-risk patients with an immune response that successfully prevents pulmonary involvement in SARS-CoV-2 infection to the response of patients developing COVID-19 pneumonia. We performed single-cell RNA sequencing (sc-RNA seq), in-depth RNA sequencing of sorted immune cell populations and nasal swabs, 50-dimensional flow cytometry, plasma shotgun proteomics together with cytokine profiling longitudinally throughout the disease course. We used a longitudinally sampled exploratory high-risk cohort for hypothesis generation (*n* = 14 patients), an independent longitudinally sampled outpatient versus hospitalized confirmation cohort for peripheral blood mononuclear cell (PBMC) and plasma analysis (*n* = 58), and nasal swabs from ambulatory as well as hospitalized patients to analyze local immune responses (*n* = 69 patients) (Fig. 1a, b, also see Supplementary Table 1 for WHO severity scale and Supplementary Table 2 for viral load data).

For the exploratory cohort, the sampling time points post PCR test positivity were day 3.0 [IQR 2.5,6.5] first sampling, median day 8.0 [IQR 8,11] second sampling and median day 17 [IQR 14,35] third sampling (Fig. 1a and Suppl. Fig. 1a, b, and Methods section).

In our exploratory cohort, radiological assessment of non-pneumonic, SARS-CoV-2 infected patients revealed no signs of lung injury on high-resolution computed tomography compared to patients experiencing pulmonary symptoms (Fig. 1c). Upper respiratory tract viral loads derived from nasopharyngeal swabs between pneumonic and non-pneumonic patients in the exploratory group were comparable (Fig. 1d). Patients in the exploratory cohort had on average 2.9 risk factors for developing a severe disease course, with all patients having at least 1 risk factor for a severe disease course, and a trend towards more risk factors in the non-pneumonic cohort (Table 1, see Methods section).

**Table 1:** Additional clinical characteristics of exploratory patient cohort.

Cohort:	Control	Non-pneumonic COVID-19	Pneumonic COVID-19	Two-sided *t* test / Fisher’s exact test non-pneumonic vs. pneumonic
Patient count	3	4	7	–
Age, median year [interquartile range [IQR]]	74 [69–74]	72 [41–88]	61 [37–75]	n.s.
Male, *n* [%]	2 [66]	4 [100]	4 [57]	n.s.
COVID-19 risk factors				
Cardiovascular disease, *n* [%]	3 [100]	3 [75]	2 [29]	n.s.
Arterial hypertension, *n* [%]	3 [100]	3 [75]	3 [43]	n.s.
Diabetes mellitus, *n* [%]	2 [66]	2 [50]	3 [43]	n.s.
Asthma/COPD/OSAS, *n* [%]	1 [33]	1 [25]	1 [14]	n.s.
Male, *n* [%]	2 [66]	4 [100]	4 [57]	n.s.
Age >60, *n* [%]	3 [100]	2 [50]	4 [57]	n.s.
≥1 risk factors for severe COVID-19, *n* [%]	3 [100]	4 [100]	7 [100]	n.s.
Pathogens (if tested)				
SARS-CoV-2, *n* [% positive result]	0 [0]	4 [100]	7 [100]	–
Other pathogens (Influenza, RSV, etc.), *n* [% positive result]	0 [0]	0 [0]	0 [0]	–
Clinical information at admission				
COVID-19 typical symptoms (New onset fever, cough or dyspnea), *n* [%]	0 [0]	2 [50]	7 [100]	n.s.
Clinical information during enrollment in the study				
O2-requirement	0 [0]	0 [0]	6 [86]	*
Immunomodulatory treatment/trial enrollment	0 [0]	0 [0]	3 [43]	n.s.
Radiological findings				
Chest CT, *n* [%]	3 [100]	3 [75]	7 [100]	
COVID-19 typical bipulmonary infiltrates, *n* [%]	0 [0]	0 [0]	7 [100]	**
COVID-19 typical ground glass opacities, *n* [%]	0 [0]	0 [0]	7 [100]	**

*p < 0.05, **p < 0.01.

Absolute leukocyte counts did not differ between groups, while other inflammatory markers such as CRP, IL-6, and Lactate Dehydrogenase (LDH) were elevated in the pneumonic group as expected (Fig. 1e). Further differentiation of leukocyte subsets revealed lower lymphocyte counts in the pneumonic group, but similar neutrophil and monocyte counts as reported previously (Suppl. Fig. 1c)^8,12,23,24^.

To allow for an in-depth analysis of leukocyte subsets, we performed phenotyping of surface marker expression by flow cytometry in our exploratory cohort (Suppl. Fig. 1d). Changes in the mononuclear phagocyte (MNP) and NK-cell compartment included no major differences at early timepoints and higher relative pDC counts in non-pneumonic patients compared to patients with pulmonary injury throughout the disease course (Suppl Fig. 1e).

We also observed shifts in T cell populations at late timepoints, mainly presenting with a relative increase in CD4^+^ T cell counts in non-pneumonic patients, but without numerical changes in CD8^+^ T cells (Suppl Fig. 1e). These trends remained if patients were binned according to time after PCR-positivity (Suppl. Fig. 1e).

Unsupervised UMAP clustering of single-cell RNA-seq data of PBMCs identified 17 distinct immune cell populations (Suppl. Fig. 2a, b). The subclusters were integrated into the following immune cell populations: T cells, B cells, monocytes, NK cells, myeloid dendritic cells, plasmacytoid dendritic cells, plasma cells, proliferating cells, and gamma delta T cells (Fig. 1f, g). T cells were further differentiated into CD4^+^ (Population 5), CD8^+^ (Populations 1, 4, 8), and γδ T cells (Population 13). The top cluster defining genes of the overarching cell types are depicted in Suppl. Fig. 2a. Consistent with our flow cytometry data, cell-based clustering revealed no major differences between cohorts (Fig. 1g).

Plasma shotgun proteomics identified 1102 proteins. Internal quality control revealed a strong positive correlation of CRP determined by our proteomic approach with clinical CRP (*r* = 0.9136) and fibrinogen measurements (*r* = 0.7967, Suppl. Fig. 3a). Principal component analysis slightly separated control patients from SARS-CoV-2 infected patients, without strong differences between disease severities (Suppl. Fig. 3b). Comparison with another plasma proteome study of COVID patients^25^ showed correlations of the plasma proteome up to 38% (Suppl. Fig. 3c). 171 plasma proteins were significantly upregulated in either the pneumonic or non-pneumonic group and downregulated in the other group in comparison to healthy controls (Suppl. Fig. 3d). Especially acute phase plasma proteins (APO-E, PZP, CSFR1, SERPINA1), coagulation factors (FCN2, F5, F11), and components of the complement system (C3, C1RL) were upregulated in pneumonic COVID-19 patients (Fig. 1h and Suppl. Fig. 3e).

Taken together, comparison of non-pneumonic and pneumonic patients revealed only mild global changes in circulating immune cells and plasma proteins, consistent with previous reports^18^.

### Enhanced interferon response across immune cell populations defines protective immunity in non-pneumonic SARS-CoV-2 infection

To better understand how the cellular response to SARS-CoV-2 infection might shape disease course, we next analyzed our transcriptomic data in detail.

**Fig. 2:**
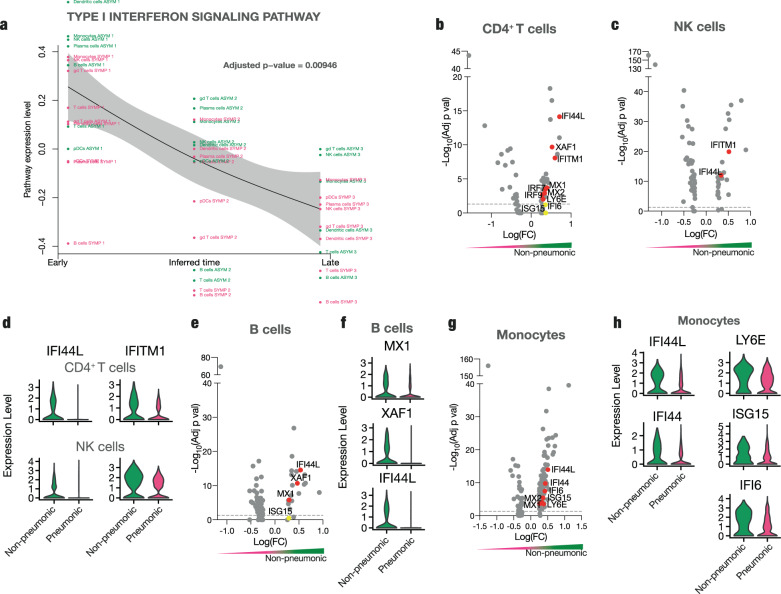
Enhanced interferon response across immune cell populations defines protective immunity in SARS-CoV-2 infection. **a** Tempora based analysis of longitudinally alternating gene pathways across cell clusters of pneumonic and non-pneumonic patients yielding “Type I Interferon signaling pathway” as a temporally significantly regulated pathway, non-pneumonic clusters are depicted in green, pneumonic clusters are in red, timepoints are chronologically set throughout the inferred time axis and depicted in the cluster names, statistical test conducted using the Tempora method. **b**, **c** Volcano plots of differentially regulated genes in CD4^+^ T cells and NK cells of pneumonic compared to non-pneumonic samples. Genes enriched in pneumonic samples have negative log(FC), genes enriched in non-pneumonic samples have positive log(FC). The color scale underneath emphasizes this (red pneumonic, green non-pneumonic) **d** Violin plots of expression of IFI44L and IFITM1 in CD4^+^ T cells and NK cells for pneumonic and non-pneumonic samples. **e** Volcano plot of differentially regulated genes in B cells of pneumonic compared to non-pneumonic samples. **f** Violin plots of expression of MX1, XAF1, and IFI44L in B cells for pneumonic and non-pneumonic samples. **g** Volcano plot of differentially regulated genes in monocytes of pneumonic compared to non-pneumonic samples. **h** Violin plots of expression of IFI44L, IFI44, LY6E, ISG15, and IFI6 in monocytes for pneumonic and non-pneumonic samples. **b**, **c**, **e**, **g** Red annotations are significantly upregulated (adj *p* val < 0.05), yellow ones are non-significantly differentially expressed. Positive fold change signifies higher expression in the non-pneumonic group. Line denotes adj *p* val < 0.05. Statistical testing for volcano plots described in methods. **b**–**h** Longitudinal samples are pooled if not otherwise indicated.

First, we sought to identify global, dynamic pathways that showed significant variation over the disease course in the exploratory cohort. We reasoned that these might be crucial in shaping disease outcome^26^. For this purpose, we utilized an algorithm that fully exploits the advantage of longitudinal sampling and allows cell trajectory inferences without relying on pseudotime analyses: Tempora^27^ deducts longitudinally alternating integrative gene pathways (further depicted in methods). Significant cellular trajectories identified across all immune cell types evolved around interferon signaling and included pathways such as “type I interferon signaling” or “interferon alpha beta signaling”. These showed strong temporal expression variation, driven mainly by high expression levels in non-pneumonic individuals at first sampling (Fig. 2a and Suppl Fig. 4a). To understand if interferon signaling indeed differs between pneumonic and non-pneumonic patients, we directly compared differentially expressed genes (DEGs) across sampling time points between the two disease states. Assessment of T cell, B cell, NK cell, and monocyte populations revealed consistent upregulation of interferon-stimulated genes (ISGs) in the non-pneumonic cohort. For example, transcripts encoding Interferon Induced Protein 44 Like (*IFI44L*) and Interferon-induced Transmembrane Protein 1 (*IFITM1*), both of which are involved in viral containment^28,29^, showed significantly higher levels in both CD4^+^ T cells and NK cells of non-pneumonic SARS-CoV-2 infected patients (Fig. 2b–d). Additionally, interferon signaling in B-cells was elevated in patients without pulmonary injury, indicated by increased expression of *MX1*^30^, *XAF1*^31^, and *IFI44L* compared to patients with pneumonic COVID-19 (Fig. 2e, f). *IFI44L*^28^, *IFI44*^28^, *IFI6*^32^, *LY6E*^32^, *ISG15*^33^ were significantly higher expressed in monocytes from non-pneumonic cases compared to patients with pulmonary involvement (Fig. 2g, h).

To further analyze longitudinal dynamics of ISG responses, we performed a focused Tempora analysis including our predetermined ISG gene list (see Methods section)^27^. This analysis of the longitudinal behavior of ISGs in non-pneumonic patients revealed a strong and early burst of ISG signaling across multiple cell clusters (Suppl. Fig. 4b).

**Fig. 3:**
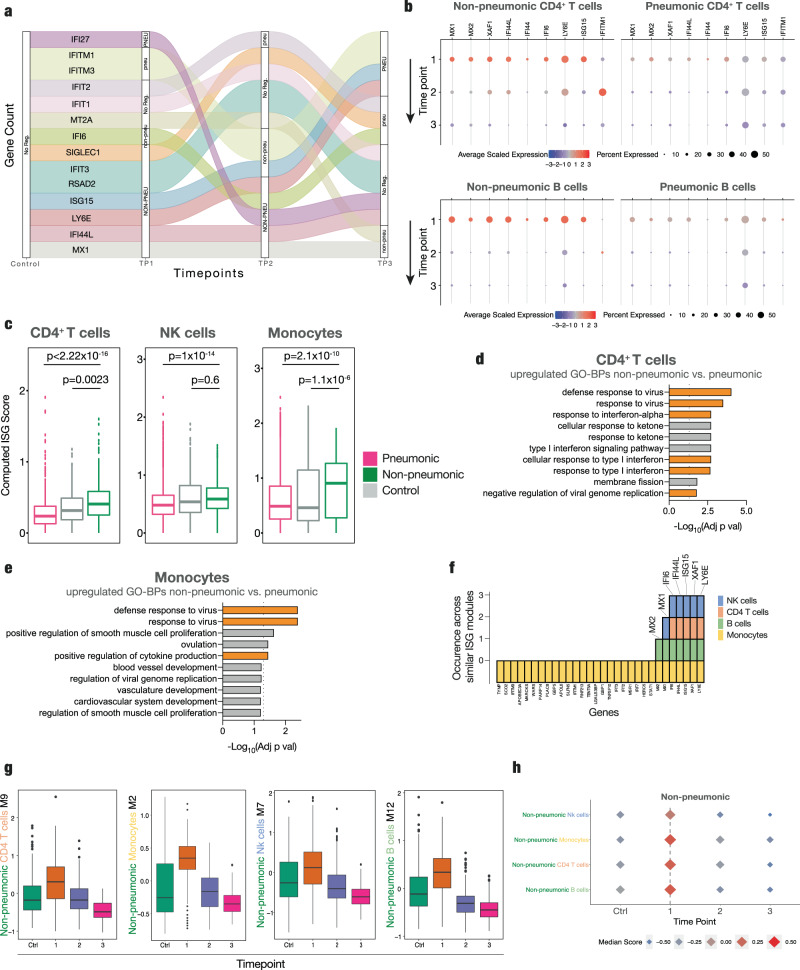
Differentially regulated interferon response across time points. **a** Double-differential time series plot of monocytes depicting which patient group shows increased expression of predefined ISGs, always in comparison to non-infected controls at baseline, binned by the 0.25 and 0.75 quantiles of all (absolute) double-differential fold changes into PNEU, pneu, No Reg, non-pneu, and NON-PNEU, which display different magnitudes of differential regulation. **b** Dot-plot of the scaled average expression and percent expressing cells of selected interferon-stimulated genes in CD4^+^ T-cells and B cells by sampling time point in non-pneumonic and pneumonic patients. **c** Box plots of ISG-scores (see methods) of CD4^+^ T cells (*n* =  725 pneumonic *n* = 388 non- pneumonic and *n* = 162 control cells), NK cells (*n* =  2550 pneumonic *n* = 888 non- pneumonic and *n* = 903 control cells) and monocytes (*n* =  1713 pneumonic *n* = 380 non- pneumonic and *n* = 1303 control cells). P values are shown above, non-pneumonic with pneumonic and control with pneumonic are compared. Box-and-whiskers plot (median, IQR, and 1.5IQR), two-sided t-tests. **d**, **e** Top 10 Gene Ontology - Biological Processes (GO-BPs) terms from upregulated genes of non-pneumonic vs pneumonic samples. Pathways of interest are marked, line shows adj *p* val < 0.05. Statistical testing described in methods. **c**–**e** Longitudinal samples are pooled if not otherwise indicated. **f** Number of identical occurrence of genes of ISG GMs of non-pneumonic patients in four immune cell subsets. **g** Box-plots showing temporal development of the specific ISG-GMs in non-pneumonic patients. Box-and-whiskers plot (median, IQR, and 1.5IQR). **h** Dot plots displaying the median score of respective ISG-modules of NK-cells, Monocytes, CD4 T cells, B cells throughout time in non-pneumonic patients and in control patients. CD4 T cells *n* = 162,155,185 and 48 per TP respectively, monocytes *n* = 1303,131,208 and 41 per TP respectively, NK cells *n* = 903,314,495 and 79 per TP respectively and B cells *n* = 440,264,279 and 103 per TP respectively. Source data are provided as a Source Data file.

We next subdivided ISGs into groups that showed similar longitudinal development across the disease course by a double-differential time series. This is depicted as an Alluvial plot with distinct color-coded trajectory groups, each characterized by a common temporal regulation – either enriched in pneumonic or non-pneumonic patients compared to controls (Fig. 3a). Timepoints were given as time point (TP) 0 for healthy controls followed by the longitudinal sampling time points of the infected patients. In monocytes, the number of upregulated ISGs at TP1 was more frequent in non-pneumonic patients and included *LY6E*, *IFI44L*, *ISG15,* or *MX1* (Fig. 3a). These ISG trajectories showed a similar subsequent course with steady higher expression in non-pneumonic patients at TP2 compared to controls (Fig. 3a).

When directly resolving ISG expression, we confirmed key interferon signaling-related genes across different immune cell subsets to be most prominently and abundantly expressed at the earliest time point of disease course in non-pneumonic patients (Fig. 3b and Suppl. Fig. 4c). This underscores the notion that ISGs play an early and potentially deterministic role in shaping COVID-19 disease course^34^. Most of the displayed genes possess antiviral activity: *MX1, MX2, IFI44, IFI 44L,* and *ISG15* inhibit viral replication^33,35,36^, *IFITM1* inhibits viral fusion^37^ and LY6E impairs coronavirus fusion^38^. In vitro studies on SARS-CoV-2 have revealed a suppressed or delayed interferon response by infected cells, mediated by multiple viral proteins inhibiting the physiological antiviral response^39,40^. We therefore asked if the observed differences simply indicated a downregulation of the interferon response in severe COVID-19. However, when comparing non-pneumonic SARS-CoV-2 infected patients with uninfected control patients, we found persistent differential expression, pointing towards an active enhancement of ISG signaling in peripheral immune cells as a key immunological feature correlating with non-pneumonic SARS-CoV-2 infection (Suppl. Fig. 4d, f–h).

To further validate these findings, we computed an ISG-score for each cell population using a modified score, based on Hadjadj et al.^12^ and Combes et al.^12,41^. The strong increase in ISGs in non-pneumonic compared to pneumonic COVID-19 patients was reflected by an elevated ISG-score across CD4^+^ T cells, NK cells and was particularly prominent in monocytes (Fig. 3c). For further validation, we used a larger ISG set to compute a broader ISG score (including 57 ISGs, see Methods section), which yielded similar results (Suppl. Fig. 4i).

Finally, gene ontology (GO) term analysis of biological processes revealed an enrichment of “defense response to virus”, “response to virus”, “response to interferon-alpha” “response to type I interferon”, “negative regulation of viral genome replication”, and several other interferon-related pathways in CD4^+^ T cells and monocytes (Fig. 3d, e and Suppl. Fig. 4e).

To better understand if the identified ISG responses represent jointly regulated gene modules on the single-cell level, we employed a framework allowing an unbiased identification of coordinated transcriptional changes across single cells and their deconvolution over the disease course. Weighted gene correlation network analysis (WGCNA), previously described to identify coordinative cellular responses in HIV infection^26^, was used for non-pneumonic SARS-CoV-2 infection and compared to pneumonic COVID-19. In brief, gene modules (GMs), depicting mutually regulated genes, were identified for the main cell clusters in non-pneumonic and in pneumonic patients (respectively pooled with healthy clusters) in an unbiased manner. Interestingly, across multiple cell subsets a distinct interferon stimulated gene (ISG) enriched GM was present. These GMs, termed ISG-GMs, were comprised of genes such as *Ly6E*, *XAF1*, *IFI6*, *IFI44L*, *MX1,* or *ISG15* (Fig. 3f). The broadest ISG-GM could be detected in monocytes, comprising more than 30 genes (Fig. 3f and Suppl Fig. 4j). Longitudinal analysis revealed these ISG-GMs to be enriched early in the non-pneumonic disease course during peak viral load, particularly in monocyte, B cell, and CD4^+^ T cell clusters (Fig. 3g, h and Suppl. Fig. 4k, l).

Together, these data point towards an early, global induction of coordinated interferon I gene modules in peripheral immune cells, correlating with the ability to successfully contain SARS-CoV-2 infection.

### Decreased cytotoxic potential of lymphocytes in non-pulmonary SARS-CoV-2 infection

To explore additional protective immune cell trajectories, we analyzed the transcriptome of lymphocytes in greater detail. Cytotoxic lymphocytes are key to both the adaptive and innate immune response to viral infections, with cytotoxic T cells and NK cells representing adaptive and innate antiviral branches respectively^42^. Prior work has highlighted upregulation of cytotoxic pathways in severe COVID-19, which could mediate harmful tissue damage^43,44^.

**Fig. 4:**
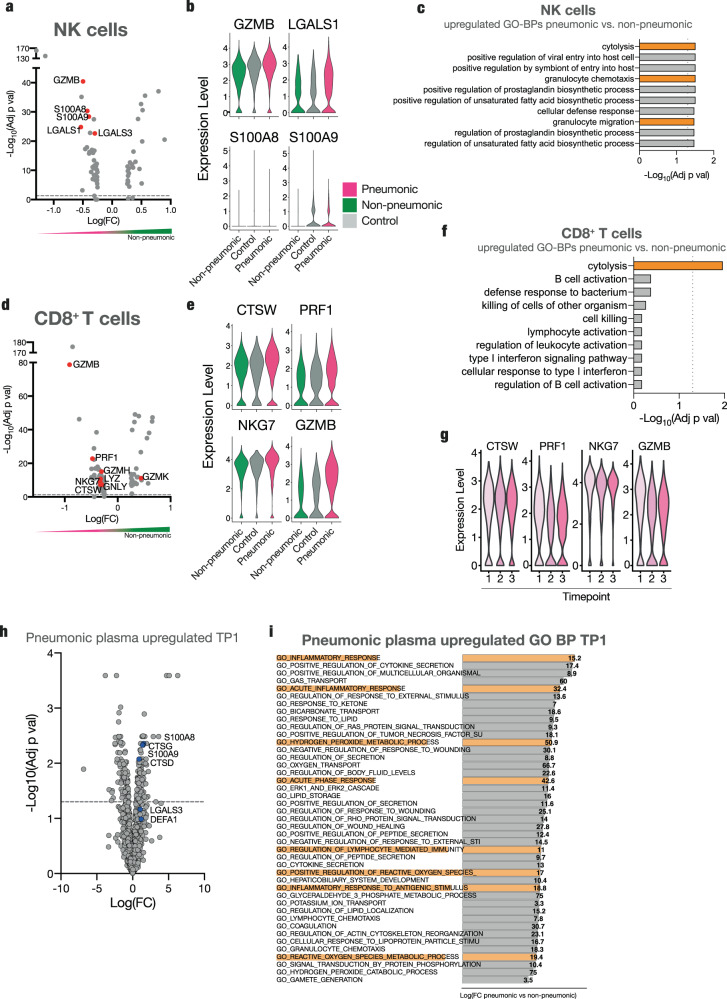
Downregulation of cytotoxic potential in lymphocytes in non-pneumonic SARS-CoV-2 infection. **a** Volcano plot of differentially regulated genes in NK cells of pneumonic compared to non-pneumonic samples. **b** Violin plots of expression of GZMB, S100A8, LGALS1, and S100A9 in NK cells for pneumonic, control, and non-pneumonic samples. **c** Top 10 GO-BPs from upregulated genes of non-pneumonic vs pneumonic samples in NK cells. Pathways of interest are marked, line shows adj *p* val < 0.05. **d** Volcano plot of differentially regulated genes in CD8^+^ T cells of pneumonic compared to non-pneumonic samples. **a**, **d** Red annotations are significantly differentially regulated (adj *p* val < 0.05. Positive fold change signifies higher expression in the non-pneumonic group. Line denotes adj *p* val < 0.05. **e** Violin plots of expression of CTSW, PRF1, NKG7, and GZMB in CD8^+^ T cells for pneumonic, control, and non-pneumonic samples. **f** Top 10 upregulated GP-BPs of non-pneumonic vs pneumonic samples in CD8^+^ T cells. Pathways of interest are marked, line shows adj *p* val < 0.05. **g** Violin plots of expression of CTSW, PRF1, NKG7, and GZMB in CD8^+^ T cells per sampling time point. **h** Volcano plot of differentially expressed plasma proteins at TP1 of pneumonic samples compared to control samples. Line denotes adj *p* val < 0.05. **i** Top significantly enriched GO-BPs for pneumonic plasma proteins compared to non-pneumonic plasma proteins. Pathways of interest to lymphocyte cytotoxicity are marked. Statistical testing for GO-BPs and volcanos described in methods. Source data are provided as a Source Data file.

NK cells of infected pneumonic patients exhibited an increase in markers of activation and cytolysis, such as granzyme B (*GZMB*)^28^, granzyme H (*GZMH*)^45^ as well as galectin-1 and galectin-3 (*LGALS1*, *LGALS3*)^46^ (Fig. 4a, b and Suppl. Fig. 5a), compared to cases without lung involvement. NK cell activation markers and neutrophil chemoattractants *S100A9* and *S100A8*^47^ were also less abundantly expressed in non-pneumonic infected patients. In line, NK cells from pneumonic patients showed an upregulation of GO terms such as “cytolysis”, “granulocyte chemotaxis”, and “granulocyte migration” (Fig. 4c and Suppl. Fig. 5b). Flow cytometry-based surface profiling revealed five NK cell subpopulations among pneumonic and non-pneumonic patient groups characterized by differential surface expression (Suppl. Fig. 5c–g). Subpopulation NK2, characterized by increased expression levels of CD18, which is necessary for cytotoxic activity^48^, and CD9, a tetraspanin implicated in innate immune cell activation and diapedesis^49^, was detected at high levels throughout pneumonic disease (Suppl. Fig. 5d, f).

Similarly, infected patients without lung injury showed a less cytotoxic CD8^+^ T cell transcriptomic phenotype with decreased expression levels of cytotoxic genes such as *CTSW*^45,50^, *PRF1*^51^, *NKG7*^47^, *GZMB*^52,53^, *GZMH*^45^, or *GNLY*^54^ (Fig. 4d). This reduced cytotoxic potential was not only observed in comparison to pneumonic COVID-19 but partially also in comparison to control subjects, indicative of a mild downregulation of cytotoxicity genes (Fig. 4d, e). Accordingly, GO enrichment analysis for biological processes revealed a relative downregulation of the cytolysis pathway in CD8^+^ T cells from patients without pulmonary injury (Fig. 4f and Suppl. Fig. 5h). Temporal resolution revealed the respective cytotoxicity genes in CD8^+^ T cells and NK cells to be expressed early in pneumonic COVID-19 patients, with a high expression throughout the disease course (Fig. 4g).

Plasma proteomic analyses were in line with decreased cytotoxic features of NK and T cells in non-pneumonic infection. *S100A8*, *S100A9,* and *LGALS3* were enriched in pneumonic plasma, especially during the first time point, whereas non-pneumonic patients did not have an enrichment of these markers (Fig. 4h and Suppl. Fig. 5i). These markers have previously been shown to be upregulated in severe COVID-19 and are associated with a severe disease outcome^55^. Other indicators of the increased cytotoxicity, cathepsin G and D, and defensin alpha 1, which is not only expressed by neutrophils, but also NK cells, were upregulated at multiple sampling timepoints in pneumonic patients (Fig. 4h and Suppl. Fig. 5i)^56,57^. Pathway analyses of differentially expressed plasma proteins also revealed upregulated inflammation, acute response, and oxygen species pathways, with the most pronounced upregulating at the first sampling point (Fig. 4i and Suppl. Fig. 5j). Indeed, cytotoxic T lymphocytes and NK cells have been shown to mediate cytotoxicity by release of reactive oxygen species^58^.

In summary, reduced upregulation of circulating lymphocyte cytotoxicity markers associate with a non-pneumonic disease course.

### Abundant naïve and immune-modulatory T-cells and antiviral NK-cells in non-pneumonic SARS-CoV-2 infection

**Fig. 5:**
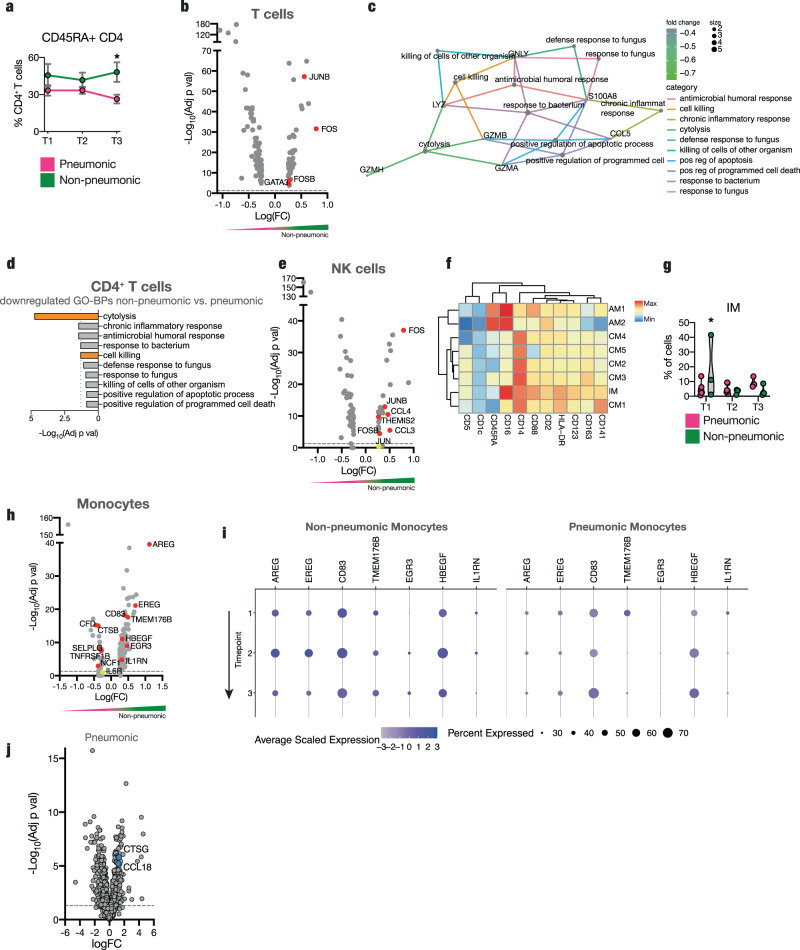
Abundant unexperienced and immune-modulatory T-cells and antiviral NK-cells and anti-inflammatory and anti-thrombotic monocytes in non-pneumonic SARS-CoV-2 infection. **a** Percentage of CD45RA^+^ CD4^+^ T cells of live PBMCs measured by flow cytometry per sampling time point. Two-sided t-test, *n* = 4 per time point for non-pneumonic, *n* = 7,4 and 7 per time point respectively for pneumonic. *p* = 0.0170. **b** Volcano plot of differentially regulated genes in all T cells of pneumonic compared to non-pneumonic samples. **c**, **d** GO-BP network analysis and Top10 downregulated GP-BPs of non-pneumonic vs pneumonic samples in CD4^+^ T cells. Pathways of interest are marked, line shows adj *p* val < 0.05. **e** Volcano plot of differentially regulated genes in NK cells of non-pneumonic compared to pneumonic samples. **f** Heat map of surface marker expression by FlowSOM group of flow cytometric measurement of monocytes. **g** Violin plot of Mono0 FlowSOM cluster per time point. Mixed-effects model analysis. Post-hoc Sidak’s multiple comparisons test for individual significant differences between pneumonic and non-pneumonic samples per time point. *n* = 3,4 and 4 per time point respectively for non-pneumonic, *n* = 7,7 and 3 per time point respectively for pneumonic. *p* = 0.0426. **h** Volcano plot of differentially up- and downregulated genes in monocytes of non-pneumonic compared to pneumonic samples. **i** Expression of selected genes from **h** in monocytes by disease condition and sampling time point. **j** Volcano plot of differentially expressed plasma proteins of pneumonic samples (all time points pooled) compared to control samples (one-sample moderated t-test). Line denotes adj *p* val < 0.05. **b**, **e**, **h** Red annotations are significantly upregulated adj *p* val < 0.05. Positive fold change signifies higher expression in the non-pneumonic group. Statistical testing for GO-BPs and volcanos described in methods. Source data are provided as a Source Data file. Line denotes adj *p* val < 0.05. All error bars are mean ± s.e.m. *p < 0.05.

In-depth analysis of CD4^+^ T cells revealed higher counts of antigen inexperienced CD45RA^+^ CD4^+^ T cells in non-pneumonic patients, most pronounced at late stages (Fig. 5a). In line, antigen experienced CD45RO^+^ CD4^+^ T cells diverged significantly during the disease course in non-pneumonic compared to pneumonic patients (Suppl. Fig. 6a).

tSNE analysis with subsequent FlowSOM subclustering of T cell panel 1 revealed a distinct CD4^+^ CD45RA^+^ CCR10^int^ naïve subpopulation (TN_1) to be significantly enriched in non-pneumonic patients, with an increased divergence over the disease course (Suppl. Fig. 6b–f and Suppl. Table 4). TN_1 was further characterized by particularly low expression of surface activation and exhaustion markers, including CXCR3, PD-1, and CTLA-4^59^ (Suppl. Fig. 6c). Interestingly, CXCR3, which is rapidly induced upon activation of naïve T cells, was expressed only at low levels in TN_1^60^ (Suppl. Fig. 6c). Further characterization by T cell panel 2 revealed a CD4^+^ CD45RA^+^ antigen inexperienced T cell population (TN_2) specific to non-pneumonic patients, corresponding to TN_1 in T-cell panel 1 (Suppl. Table 5). CD197 (CCR7) and CD27 levels in this population were intermediate, underlining a naïve phenotype^61,62^ (Suppl. Fig. 6g–k). In line with these distinct changes in surface expression, our scRNA-seq data highlighted a more immune-modulatory phenotype of T cells in non-pneumonic infected patients: *JUNB* and *FOS* -both enhanced in T cells from non-pneumonic patients- are subunits of the activating protein-1 (AP-1)^63,64^, implicated in T-cell differentiation into effector Th2^65^. *GATA3*, a master transcription factor for the differentiation of Th2 cells, was similarly upregulated (Fig. 5b)^66^. This underlines a potentially immunomodulatory or anti-inflammatory phenotype of T cells in non-pneumonic infection^67^. In line, and similar to CD8^+^ T cells, CD4^+^ T-cells from non-pneumonic SARS-CoV-2 infected patients showed downregulation of “cell killing”, “chronic inflammatory response” and “cytolysis” GO terms (Fig. 5c, d).

Corresponding to the expression profiles of T cells, the antiviral transcription factors *JUNB*, *FOS,* and *FOSB* were increasingly expressed in NK cells of non-pneumonic patients (Fig. 5e). Subcluster NK3, characterized by high L-selectin (CD62L) expression and reminiscent of an antiviral CD56^dim^ NK cell subset^68^, showed a significant increase among non-pneumonic SARS-CoV-2-infected individuals in flow cytometry (Suppl. Fig. 5d–g). This subset can produce significant amounts of IFNy and is involved in multiple antiviral tasks after restimulation and terminal differentiation^68^. Temporal analysis showed that the increased prevalence of these immunomodulatory/anti-inflammatory pathways was constant across acute disease (TP1 and TP2) (Suppl. Fig. 7a), lacking the striking early expression peak identified for ISGs (compare Fig. 3b). Comparison of non-pneumonic patients to uninfected control patients mainly recapitulated the observed differences to pneumonic patients (Suppl. Fig. 7b).

In summary, there are significantly enhanced frequencies of naïve and immune-modulatory T cells and antiviral NK cells associated with non-pneumonic SARS-CoV-2 infection.

### Monocytes with immune-modulatory potential in non-pneumonic COVID-19

As severe COVID-19 immunopathology precedes effective adaptive immune cell function, research has highlighted an innate immune axis, particularly monocytes, in COVID-19 immunopathology^8,55,69^.

What are the features defining monocytes in patients able to contain the virus in the upper airways? Flow cytometry revealed a population of non-classical monocytes characterized by surface marker expression of CD16 and CD88, as well as HLA-DR particularly early in disease (IM), (Fig. 5f, g, Suppl. Fig. 7c–f, and Suppl. Table 3). Non-classical monocytes have been implicated as crucial immune modulatory and possibly antigen-presenting cells in inflammation and infection^70^. Along these lines, CD83, which limits cytotoxic T cell effector function, was elevated on the transcriptional level in monocytes derived from non-pneumonic patients (Fig. 5h)^69,71^. Moreover, anti-inflammatory genes like *TMEM176B*, known to inhibit the inflammasome and therefore IL1-beta production, as well as Interleukin 1 receptor antagonist (*IL1RN*), showed enhanced transcription in monocytes of non-pneumonic infection (Fig. 5h)^72^.

Finally, epidermal growth factor receptor (*EGFR*) ligands, which control tissue repair and regeneration, as well as mucosal immunity, were found to be significantly enhanced in non-pneumonic cases (Fig. 5h)^73^. In particular, Ampheregulin (*AREG*), Epiregulin (*EREG*), Early growth response protein 3 (*EGR3*), and Heparin Binding EGF Like Growth Factor (*HBEGF*) showed significant upregulation (Fig. 5h). *AREG* has been shown to modulate T reg and Th2 function^74,75^, while *EREG* is crucial for epithelial integrity and resolution of inflammation^76^. Longitudinal analysis revealed that the identified pathways were most prominently upregulated at TP2, pointing to a pro-resolving and immune-modulatory monocyte phenotype developing later over the disease course (Fig. 5i). When comparing non-pneumonic patients to uninfected control patients the observed differences to pneumonic patients could essentially be recapitulated (Suppl. Fig. 7g).

In contrast, in patients developing COVID-19 pneumonia, *TNFRSF1B* and *IL6R*, genes encoding IL-6 receptor and TNF receptor, were upregulated. In addition, effector proteins like lysosomal cysteine protease Cathepsin B were significantly enhanced (Fig. 5h). Monocytes have also been implicated in contributing to immunothrombosis in COVID-19^17^. Indeed, in addition to complement factors upregulated in the plasma (Fig. 1i), complement factor D and platelet binding PSGL-1 were upregulated in monocytes of pneumonic patients (Fig. 5h). Plasma proteomics showed increased pro-inflammatory CCL18, as well as Cathepsin G (Fig. 5j)^77^.

In summary, these data highlight an overall pro-inflammatory and potentially tissue-damaging phenotype of peripheral blood monocytes in COVID-19 pneumonia. In contrast, alternative monocytes with an immune-modulatory and pro-resolving phenotype emerge in non-pneumonic patients over the course of disease.

### Robust early upregulation of ISG signatures in a large ambulatory SARS-CoV-2 infected cohort

Using a cohort of high-risk patients, we detected a distinct immune profile in non-pneumonic SARS-CoV-2 infection. This was most prominently characterized by a global ISG response across peripheral immune cell subsets, a lymphocyte shift from cytotoxic to immune-regulatory, and an anti-inflammatory monocyte signature. Longitudinal analysis highlighted an early global ISG response to be decisive for a non-pneumonic disease course, whereas the other identified immune trajectories developed over time. Because of the crucial patho-mechanistic and potentially therapeutic implications, we sought to verify our findings by focusing on ISG expression at an early time point after infection.

We chose a prospective cohort of ambulatory patients, which prospectively enrolled SARS-CoV 2 PCR-positive individuals, who were not hospitalized due to paucity of symptoms (confirmation cohort: KoCo19-Immu, see Methods)^78,79^. We included *n* = 39 PBMC samples early after RT-PCR-positivity, during acute SARS-CoV-2 infection with high nasopharyngeal viral loads (day 4), and after virus eradication and convalescence (day 60) (Suppl. Fig. 8a). Two additional cross-sectional cohorts of age-matched SARS-CoV-2 negative patients (*n* = 9) and a second hospitalized cohort with COVID-19 pneumonia (*n* = 7) were also analyzed for reference. We performed subset bulk RNA-sequencing of sorted major immune cell subsets to allow for a deep transcriptomic coverage (see Methods section).

**Fig. 6:**
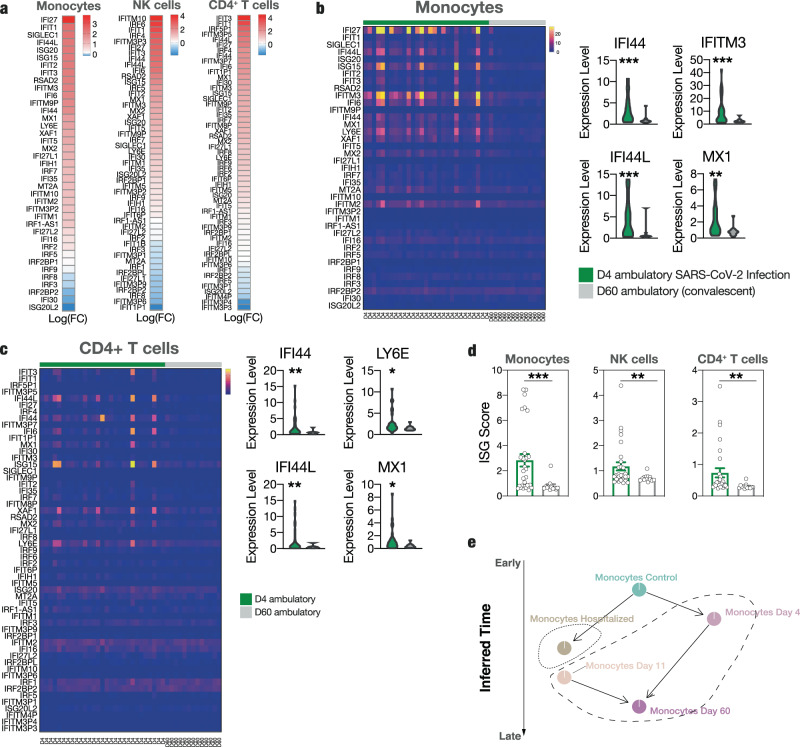
Robust early upregulation of ISGs in a large ambulatory SARS-CoV-2 infected cohort. **a** Heat maps of differentially expressed interferon stimulated genes in leukocyte subsets (monocytes, NK cells, CD4^+^ T cells) of day 4 ambulatory compared to day 60 (convalescent) COVID-19. Monocytes: *n* = 33 upregulated, *n* = 6 downregulated. NK cells: *n* = 26 upregulated, *n* = 15 downregulated CD4^+^ T cells: *n* = 44 upregulated, *n* = 7 downregulated. **b**, **c** Heat maps and violin plots of differentially expressed interferon stimulated genes in monocytes and CD4^+^ T cells of day 4 ambulatory compared to day 60 (convalescent) COVID-19. Individual ISG expressions of exemplary ISGs. Monocytes IFI44 *p* = 0.0005 IFITM3 *p* = 0.0004 IFI44L *p* = 0.0005 MX1 *p* = 0.013. CD4 T cells IFI44 *p* = 0.0066 LY6E *p* = 0.106 IFI44L *p* = 0.0070 MX1 *p* = 0.101. **d** Computed ISG scores for monocytes *p* = 0.0004, NK cells *p* = 0.0087 and CD4^+^ T cells *p* = 0.0066. **b**–**d** Unpaired two-sided t-test with Welch’s correction. *n* = 29 d4, *n* = 13 d60. **e** Tempora based analysis of monocyte trajectories from hospitalized and ambulatory patients in a longitudinal fashion. Source data are provided as a Source Data file. Mean ± sem is shown unless otherwise specified. *p < 0.05, **p < 0.01, ***p < 0.001.

In line with the exploratory cohort, ambulatory SARS-CoV-2 infected patients with oligo- to asymptomatic disease showed an early upregulated ISG signature across major PBMC subsets at day 4 compared to after convalescence at day 60 after infection (Fig. 6a). Early in the disease course, 33 ISGs in monocytes were upregulated and 6 downregulated in comparison to convalescent patients from the same cohort. (Fig. 6a). In line with our exploratory cohort*, IFI44, IFITM3, IFI44L, MX1,* and other ISGs were significantly upregulated in monocytes in ambulatory cases (Fig. 6b). Similarly, CD4^+^ T cells showed increased transcription of *IFI44*, *IFI44L*, Ly6E, and *MX1*, with overall 44 up- and 11 downregulated ISGs in CD4^+^ T cells (Fig. 6c). Transcriptional analysis of NK cells revealed upregulation of *XAF1, Ly6E, IFI6, and IFITM3*, with 36 ISGs up- and 15 downregulated altogether in NK cells (Suppl. Fig. 8b–c). Finally, the ISG score of early disease ambulatory patients was significantly increased compared to convalescent patients as reported for the exploratory scRNA Seq cohort (Fig. 6d, compare Fig. 3c). We confirmed this signature also in comparison to day 4 samples with SARS-CoV-2 negative, age-matched controls (Suppl. Fig. 8d). Furthermore, using the expanded ISG score of 57 ISGs, we further validated a significantly enhanced ISG response in early disease ambulatory patients compared to convalescent patients (Suppl. Fig. 8e).

Indeed, some ISGs were also downregulated in non-pneumonic patients compared to pneumonic patients. A more detailed insight into the downregulated genes in non-pneumonic patients, revealed a notably high number of pseudogenes (i.e. *IFITM3P4, IFITM3P3, IFITM4P, IFIT1P1* etc.) to be among the most strongly downregulated genes especially in CD4^+^ T cells and NK cells. The role of pseudogenes in the literature, and particularly ISG related pseudogenes, has not yet been conclusively resolved. Pseudogenes are largely considered not to pursue the canonical functions of their parent-gene, however some have recently been proposed to play important roles in gene regulation and silencing or functional roles unrelated to the parent-gene^80,81^. In monocytes, the downregulated genes were mostly IRFs (IRF3, 8, 9), which might indicate a negative feedback, resulting from an early ISG burst, inhibiting further IRF-mediated ISG induction. However, the downregulation of these IRFs during acute infection was very subtle as indicated in the heatmap (Fig. 6b) and presumably of minor biological relevance.

We further compared ambulatory SARS-CoV-2 infection with a cohort of hospitalized COVID-19 patients. ISG transcription in monocytes and NK-cells of day 4 ambulatory SARS-CoV-2 infected individuals was elevated compared to hospitalized patients with severe symptoms and consistent lung involvementd (Suppl. Fig. 8f, g).

For our confirmation cohort, we also reassembled the bulk immune cell data as clusters of single samples (Suppl. Fig. 8h, see Methods section). Interestingly, in comparison to ambulatory patients, immune subsets, particularly monocytes from hospitalized patients, clustered in a distinct manner (Suppl. Fig. 8h, i). Tempora^27^ analysis allows conclusions on cell type similarities in longitudinally sampled datasets and thereby enables the depiction of possible cell trajectories. Hence, we performed an unsupervised Tempora based trajectory analysis of monocyte phenotypes throughout the disease course. We set healthy patients as an early baseline throughout the inferred time and included either hospitalized or ambulatory monocytes. This allowed a better understanding of the correlation between the monocytic immune responses of both patient groups. In an unbiased manner, Tempora identified ambulatory monocyte evolution as a distinct consistent trajectory separate from hospitalized monocytes (Fig. 6e). In summary, a large, prospective, ambulatory cohort of SARS-CoV-2 infected patients validated a strong, early interferon response in peripheral blood immune cells characterizing a disease course without substantial lung involvement.

### ISG responses of the upper airway barrier surfaces do not correlate with systemic antiviral state

**Fig. 7:**
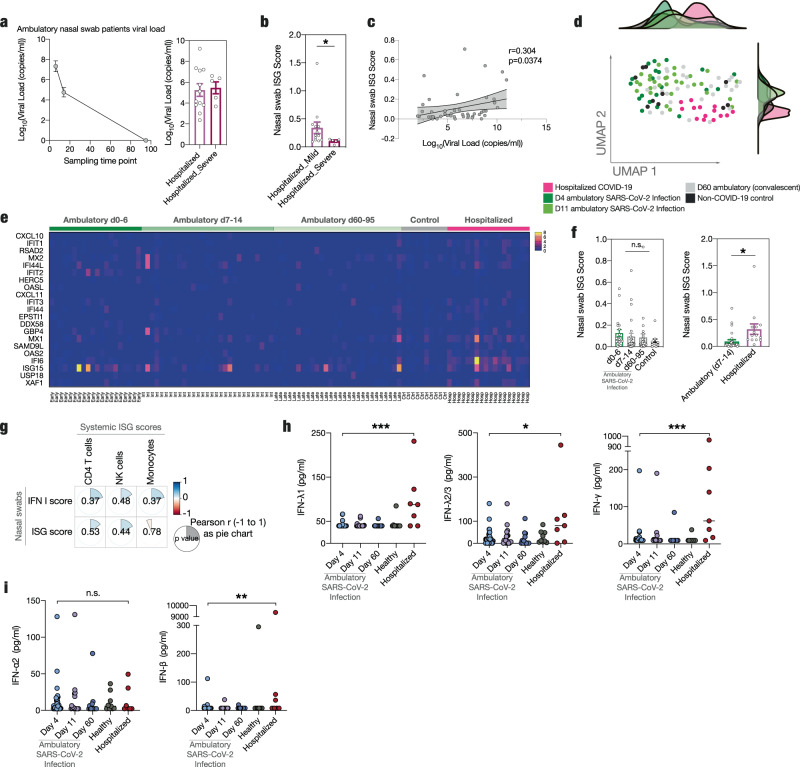
Distinct antiviral immune responses at a local vs. systemic level. **a** Longitudinal viral load course ambulatory nasal swab patients and hospitalized patients used RNA-sequencing of nasopharyngeal swab material. Log_10_(Viral Load (copies/ml) is depicted at sampling time points day 0–6 (*n* = 20), 7–14 (*n* = 29) and 60–95 (*n* = 27) post positive SARS-CoV-2 PCR (last day of sampling range used in graph). mean±sem. Hospitalized and severe hospitalized patient viral load, *n* = 12 and *n* = 5 respectively. **b** Nasal swab ISG score of nasopharyngeal swabs of hospitalized (*n* = 13) and severe hospitalized non-ICU patients (*n* = 5, see methods). Unpaired two-sided t-test with Welch’s correction, *p* = 0.0415. **c** Correlation between ambulatory d0-14 patient viral load and nasal swab ISG score. *r* and *p* value shown. *n* = 47 patients. *p*-value denotes slope non-zero. **d** UMAP clustering of patient samples. **e** Heat map of differentially expressed interferon-stimulated genes used for nasal swab ISG score. **f** Computed nasal swab ISG scores. Unpaired two-sided t-test with Welch’s correction. *p* = 0.0408 (**d**–**f**): d0-6 *n* = 20, d7-14 *n* = 29, d60-d95 *n* = 28, controls *n* = 10, hospitalized *n* = 14. **g** Pearson correlation between nasal swab ISG score and IFN I computed scores and systemic ISG scores of CD4 T cells, NK cells, and monocytes for ambulatory patients that had both early nasal swab and early blood sampling. *P* value is shown in center of each field (none <0.05). Pie charts show Pearson’s r from maximum 1 (clockwise and blue) to −1 (anticlockwise and red). *n* = 22 ambulatory patients. **h**, **i** Measurements of IFN-λ1 (*p* = 0.0003), IFN-λ2/3 (*p* = 0.0249), IFN-γ (*p* = 0.0001) (**h**), IFN-α2 (*p* = 0.8638), IFN-β (*p* = 0.0091) (**i**) in plasma samples. d4, 11 and 60 longitudinal ambulatory COVID-19 samples. *n* = 9 controls, *n* = 40 d4, *n* = 18 d11, *n* = 14 d60, *n* = 7 hospitalized COVID-19. Unpaired, two-sided Mann–Whitney *U* tests between d4 and all other groups. Non-significant results not shown, besides for hospitalized. Source data are provided as a Source Data file. Line denotes median. Mean ± sem is shown unless otherwise specified. *p < 0.05, **p < 0.01, ***p < 0.001.

Finally, we asked how the observed systemic antiviral state is induced in patients containing the infection locally. We hypothesized that a strong, early interferon response in the upper airway might prime circulating immune cells and mediate protective immunity. To investigate this further we performed RNA sequencing of nasopharyngeal swabs which allows analysis of predominantly non-immune cells such as squamous cells, secretory cells and goblet cells that are targeted by SARS-Cov-2^82,83^. We included uninfected control patients (*n* = 10), longitudinally sampled ambulatory SARS-CoV-2 positive individuals(*n* = 41) and hospitalized patients (*n* = 18 patients in total). Nasal swabs showed high viral loads in hospitalized patients as well as ambulatory patients, where the viral load gradually decreased over time, confirming adequate sampling time points (Fig. 7a). To capture the ISG response in the nasal epithelium, we used a specific ISG score derived from in vitro interferon stimulation of nasal epithelial cells for these analyses (see Methods section)^84^. Seminal work by Ziegler et al.^82^ showed abrogated upper airway ISG responses in severe hospitalized COVID-19 compared to non-severe patients, a finding that we were able to reproduce in our hospitalized cohort when we subdivided hospitalized patients based on disease severity (Fig. 7b and Suppl. Fig. 9a). We also found a positive correlation between SARS-CoV-2 viral load and ISG score, as reported in the literature (Fig. 7c)^85^.

More generally, mucosal ISG responses differentiated home-isolated SARS-CoV-2 infected individuals from hospitalized non-ICU COVID-19 cases, as UMAP showed separate clustering of hospitalized swabs (Fig. 7d).

Interestingly, early local ISG responses in ambulatory patients were not as prominently enhanced compared to time points after recovery as observed in circulating immune cells (Fig. 7e, compare Fig. 6b).

Indeed, relating local ISG responses of our ambulatory cohort to non-ICU hospitalized pneumonic COVID-19 patients revealed no positive correlation of mucosal ISG response with disease containment (Fig. 7f)^82^. On the contrary, sampling time point matched comparisons between non-ICU hospitalized COVID-19 patients and ambulatory subjects showed similar IFN type I transcription and significantly increased ISG induction in hospitalized non-ICU COVID-19 (Fig. 7f and Suppl. Fig. 9b–d). Lastly, we correlated nasal swab ISG response and IFN I score with the systemic immune cell ISG score for individuals for whom we had performed both analyses. We did not detect significant correlation between the two scores (Fig. 7g).

In summary, we conclude that the local antiviral response of the (infected) upper airway mucosa cells, in contrast to systemic ISG responses, does not positively correlate with disease containment in the upper respiratory tract.

### Cytokine profiling reveals differences in type II and III interferon plasma levels

Next, we asked whether the observed ISG signature in circulating immune cells of SARS-CoV-2 infected ambulatory patients (confirmation cohort) was due to elevated systemic interferon levels. We deployed multiplex cytokine profiling to longitudinally map circulating plasma interferons at day 4, 11 and convalescence (day 60) of ambulatory compared to hospitalized COVID-19 and non-COVID-19 control patients.

Interestingly, in contrast to identified systemic cellular ISG responses, non-hospitalized mild cases had a significantly lower levels of interferon type II and type III (IFN-λ1, IFN-λ2/3 and IFN-γ) at early timepoints of infection (d4) compared to hospitalized COVID-19 patients (Fig. 7h). However, there was no clear difference between early mild and hospitalized SARS-CoV-2 infection for interferon type I plasma levels, with no difference in circulating IFN-α2 and only slightly higher IFN-β in hospitalized patients (Fig. 7i). There was no difference between d4 and any other timepoints as well as to healthy controls. In summary, despite overt differences in ISGs, cytokine profiling provided no evidence for differences in circulating IFN levels as an explanation for the observed ISG signature across immune cell subsets in non-pneumonic SARS-CoV-2 infected patients. Indeed, the ISG scores of immune cells of hospitalized and day 4 ambulatory patients and their respective plasma interferon levels did not correlate, with no significant correlations across any cell or interferon type (Suppl. Fig. 9e).

This might indicate either a local ISG induction, for example in secondary lymphoid organs, independent of plasma-interferons, a very early transient interferon response not captured in our cohort, or interferon-independent regulation of ISG expression^86^. Plasma-interferon independent induction of cellular ISGs might explain the conflicting data currently existing on interferon responses in SARS-CoV-2 infection^13,18,19,87^.

## Discussion

The heterogeneity of responses to SARS-CoV-2 infection, ranging from non-pneumonic courses to acute respiratory distress syndrome (ARDS), holds promise for immune system modulation as a therapeutic approach. Even the majority of patients with high-risk characteristics are able to control the virus in the upper airways^88^. Here, we specifically analyzed immune responses that enable early viral containment without triggering organ damage by focusing on non-pneumonic compared to pneumonic SARS-CoV-2 infection. We utilized an at-risk cohort analyzed with state-of-the-art multi-omics assays to generate hypotheses, which we then validated in a larger prospective confirmation cohort of ambulatory infected patients^55,69^.

**Fig. 8:**
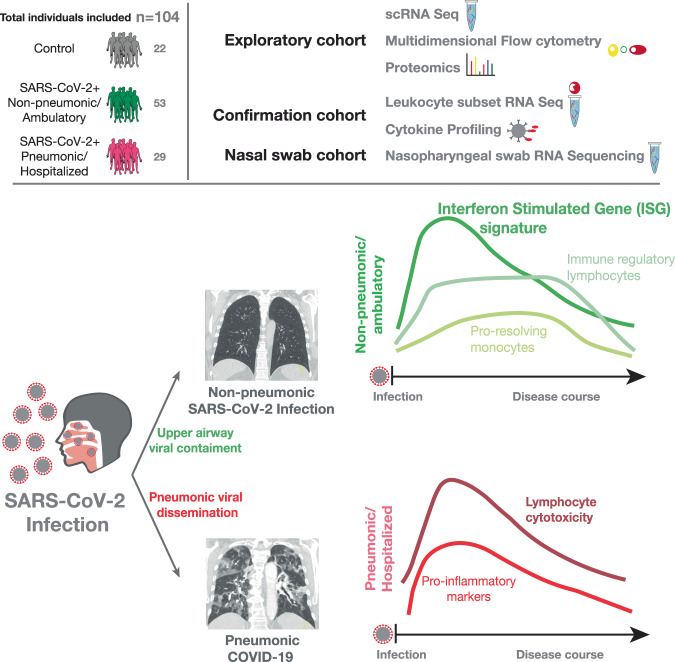
Graphical abstract. Upper left: schematic of the combined patient cohorts used. Upper right: Schematic of experimental setup. The study was divided into an exploratory cohort using scRNA-Seq, multidimensional flow cytometry of PBMCs, and shotgun plasma proteomics. The confirmation cohort was used to validate findings from the exploratory cohort, using in-depth RNA-Seq of FACS-sorted PBMCs and multiplex plasma cytokine profiling. Nasal swabs were included from both hospitalized and ambulatory patients that were either already included in the two independent cohorts mentioned above or were additionally recruited. Exact numbers for every cohort and method are depicted in Fig. 1a, b and in methods. Bottom: explanation of findings. After infection with SARS-CoV-2, the virus is either contained in the upper airway tract (“non-pneumonic SARS-CoV-2 infection”) or it disseminates into the lung (“pneumonic COVID-19”). Our study shows that non-pneumonic SARS-CoV-2 infection is characterized by an early strong interferon-stimulated-gene (ISG) signature, as well as an immune regulatory lymphocyte signature and pro-resolving monocytes in the peripheral blood. In contrast, in case of viral dissemination, pneumonic COVID-19 is characterized by lymphocyte cytotoxicity and a proinflammatory marker profile in the peripheral blood.

We observe a distinct ISG signature across multiple circulating immune cell subsets as a hallmark of early containment of SARS-CoV2 infection without the development of further complications and disease-associated hospitalization. On the single-cell level, we detect coordinately regulated ISG gene modules in peripheral blood immune cell populations early after infection by using unbiased weighted gene correlation network analysis (WGCNA)^26^. This points towards a critical role of ISG pathways in determining disease course (see Fig. 8). In addition, we recapitulate our findings in a large, prospective ambulatory confirmation study (confirmation cohort) using cell-sorted PRIME-seq technology.

In addition, we found that non-pneumonic SARS-CoV-2 infection was associated with immunomodulatory TH2 like T cell function without upregulation of cytotoxicity and a pro-resolving EGFR signature in monocytes. In combination, these trajectories could limit uncontrolled cytokine release and inflammation^73,89^. However, more research, including functional studies and assessment of Th2 associated chemokines IL-4 and IL-13, are needed to particularly address the effect of a Th2 differentiation skewing in non-pneumonic SARS-CoV-2 infection: Published data has highlighted that Th2 cells could also lead to enhanced immunopathology^90^.

In contrast, pneumonic patients showed a reduced early ISG response, but displayed an upregulation of monocyte IL6R, S100A8/A9, and an increased cytotoxicity of T cells and NK cells, which is in line with previous reports in NK cells and CD8 T cells, showing that Granzyme B expression is heightened in moderate/severe pneumonic cases^91,92^. This shift to a proinflammatory phenotype after a failed ISG response might represent a compensatory response, triggering tissue damage and explaining immunopathology and cytokine release associated with poor outcome^93^.

Conflicting data exist on systemic IFN signaling in COVID-19 depending on (1) patient collective, (2) sampling time points, and (3) disease state^13,18,19,87,94^. Very recent work even identified increased circulating type-I interferon levels in severe compared to moderate disease in addition to elevated ISG-signaling in NK cells^94^. It remains unclear which role plasma interferons and interferon-stimulated intracellular pathways play for a non-pneumonic disease course^41,82,95,96^. Our work strongly supports the hypothesis of an early pronounced, systemic ISG response as key for limiting viral spread.

So how is the observed systemic antiviral state initiated in successful viral containment? One hypothesis is an increased local innate immune response, which in turn leads to elevated IFN levels and a systemic ISG induction in immune cells. In children, increased mucosal ISG levels at baseline prior to infection mediate defense/prevention of infection in the first place^97^. Similarly, in vitro experiments show promising results for upper airway interferon priming to prevent SARS-CoV-2 infection^85^.

Interestingly, in our cohort, cytokine profiling excluded concomitant increases in circulating IFN I and III levels in oligo- and asymptomatic individuals. On the same note, we did not detect increased ISG induction in the nasal swabs of mild/asymptomatic ambulatory patients compared to pneumonic hospitalized patients. Upper airway mucosal ISGs and IFN I response did not directly correlate with systemic ISGs in our ambulatory cohort. This is in line with insights showing distinct local versus systemic immune responses during acute SARS-CoV-2 infection^95^.

Sposito et al. show low type I-IFN expression but enhanced ISGs in nasal swabs of a small cohort (*n* = 5) of home-isolated patients^96^. However, several studies investigating ISG responses in the upper airways show correlations between viral loads and ISG responses but no correlation with disease severity in non-severe patient cohorts^85,98^. An exception is severe COVID-19 – here, upper airway ISG responses are suppressed^82^, a finding that we confirm in severe, hospitalized patients. These divergent findings between ambulatory, pneumonic/hospitalized, and severe/critical disease course indicate that different mechanisms might be involved in (1) disease containment, (2) manifest spread to the lung, and (3) progression to severe disease/ARDS.

Along these lines, the fact that mild COVID-19 infection in adults is characterized by similar upper airway viral loads and infectivity compared to pneumonic patients points towards additional mechanisms beyond the first-tier immune response at the mucosal barrier surface responsible for secondary viral containment early after infection^99,100^.

By specifically investigating non-pneumonic and non-hospitalized patients, our data bridge the gap to recent insights into deranged IFN responses between different severities of hospitalized pneumonic COVID-19 patients, which are at least in part caused by inborn or acquired defects in interferon signaling. This might hold important therapeutic and diagnostic implications. Measuring systemic ISG response in early disease might help to differentiate favorable outcomes from severe disease courses, but this needs further evaluation in prospective studies.

Limitations of our study include a comparably small exploratory patient cohort. However, we confirmed our major findings in a second, independent patient cohort. Also, we only had nasal swabs of hospitalized patients available at a rather late timepoint (median day 11) without the opportunity for longitudinal analysis. Additional studies performing longitudinal sampling of respiratory tract specimens in prospective, community-based studies are necessary to further pin down the interplay of mucosal immunity with systemic immune responses.

In summary, we provide a large-scale, integrative, and longitudinal multi-omics-based analysis of immune responses focusing on ambulatory/non-pneumonic patients with successful upper airway containment. We reveal early, prominent ISG signatures cooperatively expressed on the single-cell level across circulating immune cell subsets, without correlation with local mucosal tissue responses, as the defining immune feature of uncomplicated, non-pneumonic SARS-CoV2 infection.

## Methods

### Ethics

In accordance with the Declaration of Helsinki and with the approval of the Ethics Committee of Ludwig-Maximilian-University Munich, informed consent of the patients or their guardians was obtained. COVID-19 patients are part of the COVID-19 Registry of the LMU University Hospital Munich (CORKUM, WHO trial ID DRKS00021225). Pseudonymized data was used for analysis, the CORKUM and KocoImmu studies were approved by the ethics committee of LMU Munich (No: 20-245 & No: 20-371 respectively). There was no participant compensation.

### Cohorts

We analyzed *n* = 97 PBMC samples, *n* = 124 plasma samples, and *n* = 105 swabs from a total of 104 individual patients.

Two independent cohorts were used for PBMC/plasma analyses, an exploratory cohort (scRNA-Seq, flow cytometry, plasma proteomics) and a confirmation cohort (leukocyte subset in-depth RNA-Seq, cytokine assay). The exploratory cohort was included for hypothesis generation. This was subsequently validated by the confirmation cohort, consisting of PBMC/plasma analyses from independent patients.

In addition, we included nasal swab analyses from both hospitalized and ambulatory patients that were either already included in the two independent cohorts mentioned above (*n* = 37) or were additionally recruited (*n* = 32).

#### Exploratory cohort

In total, 14 subjects were included in our exploratory cohort (*n* = 11 patients with positive SARS-CoV-2 RT-PCR and *n* = 3 non-COVID-19 control subjects). COVID-19 patients were sampled longitudinally, and three time points were included: median day 3.0 [IQR 2.5,6.5] first sampling, median day 8.0 [IQR 8,11] second sampling, and median day 17 [IQR 14,35] third sampling. 14 patients were included for flow cytometric analysis, 12 patients were included into single-cell RNA-Seq assays. Patients with severe pre-existing kidney or liver dysfunction, severe autoimmune diseases, chronic infection, patients requiring ECMO therapy, with a known coinfection with Influenza or Respiratory Syncytial Virus (RSV) were excluded. COVID-19 patients were divided into patients without any pulmonary symptoms or radiological infiltrates and patients with confirmed COVID-19 associated pneumonia. Furthermore, control subjects without COVID-19 were included. Average number of risk factors was calculated based on individual risk factor sum. Risk factors were medical or physiological conditions associated with severe COVID-19: Age > 60 years, arterial hypertension, cardiovascular disease, chronic respiratory disease, diabetes mellitus, and male gender.

#### Confirmation cohort

The confirmation cohort consisted of a total of 58 subjects included for circulating blood leukocyte subset and cytokine assays. Of these, *n* = 42 were SARS-CoV-2 positive, non-hospitalized individuals. These patients participated in the longitudinal KoCo19-Immu cohort, which enrolled SARS CoV-2 infected individuals shortly after PCR confirmation. Comprehensive longitudinal blood sampling as well as nasopharyngeal swabs were performed by household visits of field teams. Additionally, PBMC samples from *n* = 7 hospitalized COVID-19 patients on normal wards were used, as well as PBMC samples from *n* = 9 control patients without COVID-19.

For leukocyte subset RNA Seq and cytokine profiling, the ambulatory SARS CoV-2 infected individuals were analyzed at three time points after initial RT-PCR confirmed COVID-19 infection: first at day 4 after RT-PCR, second at day 11 and third at day 60 after positive RT-PCR. The median day for the first timepoint was on day 6 after symptom onset [IQR: 5 to 9.75 days], the median for the second was on day 15 [IQR: 11.75–17.25] and for the last visit on day 68 [IQR: 63.25–83.25] after symptom onset. The hospitalized COVID-19 patients in this sub-cohort were analyzed at median day 5 after positive RT-PCR or symptom onset [IQR: 3.0–15.0]. A subset of *n* = 40 longitudinally sampled, ambulatory patients were used for plasma cytokine analysis, with *n* = 40 d4, *n* = 18 d11, *n* = 14 d60 samples measured. Another subset of *n* = 39 ambulatory patients were used for subset RNA sequencing, with *n* = 39 d4, *n* = 13 d60 samples used. In addition to these 42 total ambulatory patients, a reference cohort of SARS-CoV-2 (*n* = 9) negative individuals (female: 78%, median age: 27) and hospitalized COVID-19 (*n* = 7) were recruited for the subset RNA seq and cytokine assays (female: 29%, median age: 82). The confirmation cohort was sampled and processed completely independently from the exploratory cohort to reduce any systemic bias. The KoCo19-Immu-study is conducted under the framework of the prospective population-based Koco19 cohort^72,73^. The upper respiratory tract viral load of the ambulatory cohort at day 4 was median 5.3 [IQR: 3.2, 7.0] log_10_(Viral load (copies/ml)), and of hospitalized patients median 5.7 [IQR: 3.1, 7.0]. A viral load time course of the ambulatory patients is depicted in Supplementary Fig. 8a.

#### Nasal swab samples

For nasal swab analysis the cohort included a total of 69 patients. Nasal swabs were included from both hospitalized and ambulatory patients that were either already included in the two independent cohorts mentioned above (*n* = 37) or were additionally recruited (*n* = 32). *n* = 18 were hospitalized patients with COVID-19 on normal wards (female: 39%, median age: 61), *n* = 41 were ambulatory patients (female: 54%, median age: 36) and *n* = 10 were SARS-CoV-2 negative controls (female: 20%, median age: 25, age of 2 subjects was unknown). The hospitalized patients were sampled at median day 11 [IQR: 6.5, 12] (hosp_sev 12.0 [IQR:11.0,13.0], hosp_normal 10.0 [IQR: 5.0, 12.0]) after symptom onset or positive RT-PCR and were hence similar to the 7–14 timepoint of ambulatory patients, which also were sampled at median day 11. The ambulatory patients were sampled at days 0–6, 7–14, as well as 60–95. The upper respiratory tract viral load of the ambulatory cohort for nasal swabs at day 0–6 was median 8.3 [IQR: 5.3, 8.9] log_10_(Viral load (copies/ml)), and of hospitalized patients median 5.1 [IQR: 3.8, 6.6]. A viral load time course of the ambulatory nasal swab patients is depicted in Fig. 7a.

To further subdivide hospitalized nasal swab patients on non-ICU wards, a clinical COVID-19 severity score was used^24,101–106^. Cut offs (D-Dimer > 1000 ng/ml, CRP > 100 mg/L, LDH > 245 U/L, Troponin > 18 ng/L, Ferritin > 500 µg/L, CPK > 350 U/L, IL-6 > 30 pg/ml, lymphocytes < 800/µl) were used to stratify patients according to disease severity. Patients with a score of ≥5 were classified as severe hospitalized patients on non-ICU wards.

### Peripheral blood mononuclear cell, plasma collection, and storage

For plasma isolation, heparin anticoagulated blood was centrifuged for 10 min at 650×*g* and the plasma layer was collected. Red and white blood cells were diluted at a ratio of ~1:2–1:3 with PBS. 35 ml of PBS-Blood suspension was slowly transferred on top of a 15 ml Pancoll (PAN Biotech; Cat.: P04-60500) solution to create a blood layer and subsequently centrifuged for 40 min at 700 x g with the slowest acceleration and deceleration program. The buffy coat was isolated and washed twice with PBS (at 600×*g* for 7:30 min), resuspended in RPMI, counted, washed again with PBS and then frozen at a concentration of 1 × 10^7^ cells/ml in freezing medium (90% FCS + 10% DMSO). For the confirmation cohort CPDA (Citrat-Phosphat-Dextrose-Adenin) blood was centrifuged at 1285×*g* for 10 min. Plasma was removed and stored subsequently and twice the amount of PBS was added to the cell pellet. PBS/Blood suspension was added to Leucosept tubes (Greiner) with a Ficoll-Paque at a 1:2 ratio and centrifuged at 800×*g*, the PBMC fraction was isolated subsequently. Cells were slowly frozen in a Mister Frosty for 24 h at −80 °C, and then transferred to a liquid nitrogen tank.

### PBMC processing and preparation for flow cytometry and FACS/Sorting

PBMC vials were thawed at room-temperature for 10 min and transferred to 5 ml PBS with 1% bovine serum albumin (BSA). Cell suspension was centrifuged at 400×*g* for 15 min at 4 °C. Hereafter, cells were resuspended in PBS with 1% BSA and stained on ice with respective antibody master-mix panels for subsequent flow cytometry analysis and FACS-sorting.

### FACS/Sorting for single-cell RNA-seq

Cells were stained with SYTOX™ Red (Cat No. 1936399, Invitrogen) prior to sort. CD45^+^ living singlets were FACS/sorted and centrifuged at 400×*g* for 10 min at 4 °C. The cell concentration was adjusted to 800 cells/μl in PBS.

### Single-cell RNA-seq

The Chromium Next GEM Single Cell 3’ Reagent Kit with Feature Barcoding technology (CG000206 Rev D) was used. For feature Barcoding, patient samples were tagged by TotalSeq™ anti-human Hashtag Antibodies (B0251, A0252, A0253). Hashtag antibodies were included into the antibody panel for FACS/Sorting. Three patient samples from one timepoint and group were pooled per library. In all, 5 × 10^3^ cells per sample and 15 × 10^3^ cells in total were loaded per channel. In brief, according to the manufacturer’s instructions, first Gel Beads-in-emulsion (GEMs) were generated, reverse transcribed, cleaned up and cDNA was amplified. After cDNA generation and amplification, cDNA was quality controlled and quantified. Subsequently, the 3′ gene expression library and the cell surface protein library was constructed. Sequencing was performed with Illumina NovaSeq (library preparation and sequencing was performed by IMGM laboratories).

### Single-cell RNA-seq data processing

The raw reads were obtained from the sequencing facility. A total of 12 samples were processed using cellranger 4.0.0 with the 10X human reference data GRCh38 2020A.

The resulting filtered count matrices were loaded using Seurat 3.2.1^107^, filtered (nFeature_RNA > 200, nFeature_RNA < 6000, nCount_RNA > 1000, percent.rp < 40, percent.mt < 15) to exclude doublets and dead cells, normalized (SCTransform^108^) and integrated (according to Seurat’s SCTransform integration vignette) into one combined Seurat object. On the combined Seurat object PCA was performed with default parameters and subsequent calculation of the UMAP embedding (using the first 30 PCs). FindNeighbors was called with default parameters. Seurat’s FindClusters was called with a resolution of 0.5. Gene expression was quantified for each cluster’s marker genes, which were determined by Seurat’s FindMarkers function using the t-test. Using the cluster marker genes and gene expression results cell types were predicted using the scRNA-seq cell type prediction^109^ on PanglaoDB’s marker genes^110^ and restricted to an ‘Immune system’ context. After manual curation of the predictions by the experimentalists, a cell type was assigned to each cluster. It should be noted that for all these manual curations, the finally selected cell type was the second highest scoring prediction. Disease states were split by libraries, Hashtags were not required for further disease state identification. Gene set enrichment analysis (overrepresentation analysis) on Gene Ontology (Biological Process aspect)^111^ was performed using clusterProfiler^112^. The interferon score was calculated as mean over the normalized expression values of the interferon genes shown below for each cell. For the interferon score, we first used a score that was based on Hadjadj et al.^12^ and Combes et al.^41^ using a selection of their interferon stimulated genes. To confirm this score, we used a larger set of interferon-stimulated genes including 57 interferon-stimulated genes (see lists below). In order to fully capture the ISG signature of nasal epithelia, all comparisons regarding nasal swabs were made with a new ISG score derived from a data-set of IFN stimulated nasal epithelial cells^84^. The score was formed of all genes that could be detected in the dataset and that had a >3.5 logFC upregulation after IFNα stimulation according to Giovannini-Chami et al.. Significance between groups is calculated using the t-test with ‘ggpubr’ (https://CRAN.R-project.org/package=ggpubr) stat_compare_means function. The full analysis script is available from GitHub at https://github.com/mjoppich/covidSC in the analysis_final_analysis.Rmd script. This repository also contains all scripts used for set enrichment. Functions for custom plots are included in the main analysis script.

Included genes for the interferon score: *IFI27, IFITM3, IFI44, ISG15, IFI44L, LY6E, IFIT1, MT2A, IFIT2, MX1, IFIT3, RSAD2, IFITM1,* and *SIGLEC1*.

For the large ISG set score, we included *MX1, IFITM3P2, ISG20L2, MX2, IFITM3P9, ISG20, MT2A, IFI27L2, XAF1, RSAD2, IFIT5, LY6E, SIGLEC1, IFITM1, IFITM2, IFIT1, IFIH1, IFITM3P6, IFI44, IFI16, IFITM3P3, IFI27, IFI35, IFIT6P, IFITM10, IFI27L1, IRF2BP1, IFIT3, IRF6, IRF5P1, IFI6, IRF7, IRF2, IFIT1P1, IRF9, IRF1, IFIT2, IRF5, IRF8, IFITM9P, IRF4, IFITM3P7, IFI30, IRF2BP2, IFITM4P, IFITM3, IRF2BPL, IFITM3P1, IFI44L, IRF3, IFITM3P8, IFIT1B, IRF1-AS1, IFITM5,* and *ISG15*.

Detectable IFN I genes for the IFN I score for nasal swabs included *IFNA6, IFNA10, IFNA13, IFNA14, IFNA21, IFNK*, and *IFNA6*.

Nasal swab ISG score derived from Giovannini-Chami et al. included *CXCL10, IFIT1, RSAD2, MX2, IFI44L, IFIT2, HERC5, OASL, CXCL11, IFIT3, IFI44, EPSTI1, DDX58, GBP4, MX1, SAMD9L, OAS2, IFI6, ISG15, USP18,* and *XAF1*.

### Peripheral blood mononuclear cell processing for subset RNA-seq

PBMCs were thawed at 37 °C for 5 min, added to 5 ml PBS with 1% BSA, centrifuged at 350×*g* for 10 min at 4 °C. The supernatant was removed and resuspended in 150 μl PBS with 0.5% BSA. 50 μl was added to 50 μl of panel 1 and panel 2 respectively and incubated on ice for 15 min. Probes were filtered through a 50-μm mesh and 0.1 μl of Sytox Red (Cat No. 1936399, Invitrogen) was added to the probe prior to Sort. CD4^+^, CD8^+^ T cells, NK cells and monocytes were sorted. 500–1000 cells were sorted into 100 μl Buffer RLT Plus (+1% Mercapto-Ethanol). To sort NK cells and monocytes, we used PE CD3 and CD20 (BioLegend #300308, #302306), FITC CD14 (BD Biosciences #557153), PE-Cy7 CD16 (BD Biosciences #557744), and APC-Cy7 CD56 (BioLegend #362512). To sort T cells, we used PE CD3 (BioLegend #300308), PE-Cy7 CD4 (BioLegend #357410), AF488 CD8a (BioLegend #301024), and APC-Cy7 CD19 (BioLegend #363010).

### Nasal swab preparation

For nasal swabs, upon arrival in the laboratory, a part of each nasal swab sample was aliquoted and stored at –80 °C in temperature-controlled biobank freezers until further analysis. RNA isolation from nasal swab samples was performed with the automatic extraction system TANBead® Nucleic Acid Extraction Kit OptiPure Viral Bulk Plate (W665A10, TanBead) in a Maelstrom Extractor robot (TanBead) following the manufacturer’s recommendations. Real Time RT-PCR was performed using Allplex™ SARS-CoV-2/FluA/FluB/RSV Assay (RV10259X, Seegene) in a STARlet IVD Workstation (Seegene), which allows the simultaneous detection of SARS-CoV-2 (N gene), SARS-CoV-2 (RdRP gene) and SARS-CoV-2 (S gene) together with an endogenous and exogenous control, providing further controls including human cell content of the swab.

### Library preparation and subset RNA-seq

RNA-sequencing was performed using prime-seq. A step-by step protocol can be found on protocols.io (10.17504/protocols.io.s9veh66).

Briefly, of the 1000 cells that were sorted in the lysis buffer, 50 µL (500 cells) were used to prepare RNA-seq libraries. The lysate was treated with Proteinase K (AM2548, Life Technologies), isolated with cleanup beads (GE65152105050250, Sigma-Aldrich) (2:1 beads/sample ratio), and then DNase I (EN0521, Thermo Fisher) digested. The RNA was then reverse transcribed with 30 units of Maxima H- enzyme (EP0753, Thermo Fisher), 1x Maxima H- Buffer (EP0753, Thermo Fisher), 1 mM each dNTPs (R0186, Thermo Fisher), 1 µM template-switching oligo (IDT), 1 µM barcoded oligo-dT primers (IDT) in a 10 µL reaction volume at 42 °C for 90 min. The samples belonging to the same tissue were then pooled and cleaned using cleanup beads (1:1 beads/sample ratio), resulting in 4 pools, one for monocytes, NK cells, CD4 T cells, and CD8 T cells. Following cleanup, remaining primers were digested with Exonuclease I (M0293L, NEB) at 37 °C for 20 min followed by 80 °C for 10 min. The Exonuclease I digested samples were then again cleaned using cleanup beads (1:1 beads/sample ratio).

Second strand synthesis and pre-amplification was performed using 1X KAPA HS Ready Mix (07958935001, Roche) and 0.6 µM SINGV6 primer (IDT) in a 50 µL reaction. The PCR was cycled as follows: 98 °C for 3 min; 12 cycles of 98 °C for 15 s, 65 °C for 30 s, 72 °C for 4 min; and 72 °C for 10 min. The samples were then cleaned using cleanup beads (0.8:1 beads/sample ratio) and then eluted in 10 µL of DNase/RNase-Free Distilled Water (10977-049, ThermoFisher). The Quant-iT PicoGreen dsDNA Assay Kit (P7581, Thermo Fisher) was used to quantify the amount of cDNA present, and the High-Sensitivity DNA Kit (5067-4627, Agilent) was used to qualify the size distribution.

Following QC, 2.5 µL of cDNA (2.25–5.75 ng) from each sample was used to make libraries with the NEBNext Ultra II FS Library Preparation Kit (E6177S, NEB), primarily following manufacturer’s instructions but with a five-fold lower reaction volume. Fragmentation was carried out using the supplied Enzyme Mix and Reaction buffer in a 6 µL reaction. The adapters were ligated using the supplied Ligation Master Mix, Ligation Enhancer, and a custom prime-seq Adapter (1.5 µM, IDT) in a reaction volume of 12.7 µL. Following ligation, the samples were double-size selected using SPRI-select Beads (B23317, Beckman Coulter), with 0.5 and 0.7 ratios. The samples were then amplified using a library PCR using Q5 Master Mix (M0544L, NEB), 1 µL i7 Index primer (Sigma-Aldrich), and 1 µL i5 Index primer (IDT) using the following setup: 98 °C for 30 s; 13 cycles of 98 °C for 10 s, 65 °C for 1 m 15 s, 65 °C for 5 m; and 65 °C for 4 m. A final double-size selection was performed as before using SPRI-select Beads.

After checking the concentration and quality using a high-sensitivity DNA chip (Agilent Bioanalyzer), the libraries were 150 bp paired-end sequenced on a S4 or a S1 flow cell of a NovaSeq (Ilumina). On average, ~1 × 10^7^ reads were acquired per sample for the immune cell subsets and 5 × 10^6^ for the nasal swabs.

The data was initially checked using fastqc (version 0.11.8^113^). Cutadapt (version 1.12^114^) was then used to remove any regions on the 3′ end of the read where the sequence read into the polyA tail. Following processing of the data, the zUMIs pipeline (version 2.9.4d, Parekh et al., 2018) was used to filter the data, using a phred threshold of 20 for 4 bases for both the UMI and BC, map the reads to the human genome (GRCh38) with the Gencode annotation (v35) using STAR (version 2.7.3a), and count the reads using RSubread (version 1.32.4)^115,116^.

### Subset RNA-seq data analysis/nasal swab RNA-seq data analysis

Count matrices for each cell type and two counting methods were received. The count matrices with exon counts (exon) and combined intron+exon counts (inex) were extracted for each cell type using the ‘all’ slot (non-downsampled) from zUMI’s data object. The samples and conditions were annotated with the sample names. Both count matrices were processed analogously and differentially expressed genes were determined with DESeq2 (v1.30)^117^. For the subsequent data analysis, the exon-count results are used with an adjusted (Benjamini-Hochberg) p-value cut off at 0.05. Gene set enrichment analysis (overrepresentation analysis) on Gene Ontology (Biological Process aspect) was performed using clusterProfiler (v3.18.1)^111,112^. The nasal swab samples were processed additionally using edgeR (v3.32.1) and limma (v3.46.0)^118,119^.

### Tempora analysis

For a longitudinal analysis of the scRNA-seq data we employed Tempora version 0.1.0^27^ (https://github.com/BaderLab/Tempora). The analysis was performed on the June_01_2021 release of the Human GOBP AllPathways which is made available by the Tempora authors via the Bader Lab pathway gene set database. We added our ISG gene signature consisting of *MT2A, ISG15, LY6E, IFIT1, IFIT2, IFIT3, IFITM1, IFITM3, IFI44L, IFI6, MX1, IFI27, RSAD2, SIGLEC1* to this database. We slightly adapted the plotting routines of Tempora to include the actual cluster names in the plots instead of the (required) cluster indices in both the temporal trajectories and the varying pathway plots.

We ran Tempora on the RNA assay of the integrated scRNA-seq object. Timepoints were given as timepoint 0 for control and timepoints 1, 2, and 3 as given by the samples.

Likewise, we imported the subset RNA-seq samples into Seurat (see single sample analysis (subset/nasal swab RNA-seq data) section below) and processed these using Tempora as well on a per cell type basis. Cells were grouped by cell type and timepoint. The timepoint order was given as Ctrl, Day 4, Hosp., Day 11 and Day 60.

### Time series plots (scRNA-seq data)

In a more explicit driven analysis, we calculated the differential genes between the control cells and each of the timepoints for each cell type and all cells (TPx/TP0 for x in 1,2,3). We then binned the resulting DE genes according to their logFC into 5 bins (DOWN, down, No Reg, up, UP). The borders of the bins are defined by the 0.25 and 0.75 quantiles of all absolute fold changes. Genes with an adjusted *p*-value > 0.05 or an absolute logFC < 0.25-quantile of all absolute fold changes are classified as “No Reg”.

Genes were grouped by their distinct path through the bins at the three timepoints, resulting in the different groups of genes. The plot can be restricted to groups with a minimum of contained genes (e.g. 10). Furthermore, selected genes can be highlighted (black lines) in order to understand their behavior. The gene count threshold is not applied to such genes.

TP0 (non-infected controls as baseline) shows no regulation by definition. The bin change between two timepoints can be seen as the change in differential expression between the two timepoints.

In order to display the differences between two conditions (e.g. pneumonic and non-pneumonic) a double-differential analysis was performed. The log-fold changes (in comparison to the TP0 defined baseline control patients) of the pneumonic time series were subtracted by those of the non-pneumonic time series. Again, the resulting double-differential fold changes were binned by the 0.25 and 0.75 quantiles of all (absolute) double-differential fold changes into PNEU, pneu, No Reg, non-pneu and NON-PNEU, depicting different magnitudes of upregulation either in pneumonic or non-pneumonic patients. The double-differential fold change was calculated for all genes which appear at least once significantly regulated in the time series, no further significances were considered.

### Single sample analysis (subset/nasal swab RNA-seq data)

The exonic UMI-counts from zUMIs pipeline were extracted and used as input for Seurat (4.0.2). After loading all sample counts, the Seurat object was log-normalized, and 2000 variable features were detected. After scaling and calculating 30 principal components, the UMAP algorithm was applied to these. From the PCA, first neighbors were calculated, which are subsequently used to cluster the samples at a resolution of 0.5.

### Temporal gene module detection

We performed temporal gene module detection using the method and script provided by Kazer et al.^26^. In brief, this method takes the gene expression values of the genes represented by the first few principal components of the scRNA-seq object as input for WGCNA (v1.69) functions. Here, we chose the maximal 500 genes reported by the PCASigGenes function of Seurat for each of the first eight PCs. The resulting adjacency matrix (created with soft power 7 and minimal module size 3) is transformed into a TOM and hierarchically clustered. These clusters are merged if not too dissimilar. After testing the modules for their significance (*p*-Value threshold of 0.05, 10 bins and 100 permutations), the remaining modules are tested regarding the temporal variation (sample size is minimum of module size and 150, 1000 tests, order of time points 0 < 1 < 2 < 3, *p*-Value threshold of 0.05). The remaining modules are added to the Seurat object using the AddModuleScore function (ctrl set to 5) and are reported for further visualization and discussion.

### Flow cytometry

After sample preparations for scRNA-seq and flow cytometry, 5 0µl of the cell suspension was incubated for 20 min on ice with 50 µl of the respective antibody panel, at 1:100 dilution for each antibody. After centrifugation at 400 g for 7min and resuspension in 200 µl 1% BSA with PBS, 0.2 µl of SYTOX™ Blue (Cat No. 2192317, Invitrogen) was added for live/dead staining. To avoid batch effects all samples were measured during one experimental run.

Measurements were done on a BD LSRFortessa Flow Cytometer with BD FACSDiva v9.0 software. Analysis was done using FlowJo Software (FlowJo v. 10.6.1, BD). The gating strategy used is shown in Suppl. Fig. 1d. After a common gating strategy, each cell population/antibody panel was gated separately. *n* = 7 pneumonic, *n* = 4 non-pneumonic COVID-19 and *n* = 3 control patients were used. All three different time points for the COVID samples were measured. For t-SNE and FlowSOM analysis, cell populations were downsampled using the downsample v3 plugin for FlowJo to 2000 cells per time point and sample where possible and subsequently concatenated. FlowSOM clustering with the FlowSOM v2.5 plugin^120^ was performed on the concatenated file with *n* = 5 clusters for the NK cell panel and *n* = 8 clusters for both T cell panels and the monocyte panel. The FlowSOM output used is the minimum spanning tree, heatmap of surface maker expression by FlowSOM cluster and the the percentage of cells in each cluster per group and time point. For tSNE and FlowSOM only surface markers that were not previously used for positive cell identification were used. Cell clusters were manually assigned to know sub-populations for Monocytes and T cells. Monocytes were labeled intermediate (IM, CD16hiCD14hi), classical monocytes (CM1-5, CD14hiCD16low) and alternative monocytes (AM1-2, CD16hiCD14low) (see also Supplementary Table 3). T cells were labeled CD4 T cells, CD8 T cells, double positive (DP) or double negative (DN) cells. For panel 1, Th2 (CCR4hi), TH1/17 (CCR6 hi) naïve (CD45RA hi) T cells cells were defined. For panel 2, naïve (CCR7hi CD45RAhi), central (CCR7 hi CD45RA low) and effector memory (CD45RAlow CCR7low) and TEMRA (CCR7 low CD45RA hi) cells were defined (see also Supplementary Tables 4 and 5). All antibodies used for flow cytometry are shown in Supplementary Tables 6–9.

### Plasma sample preparation for mass spectrometry and analysis

Samples were boiled in SDS-buffer and subjected to SP3-based cleanup and tryptic digest as described^121^. The method was adapted in-house to work on an AssayMAP Bravo Protein Sample Prep Platform (Agilent). A spectral library was generated by measuring 52 fractions of a high-pH fractionation using a uniform peptide mix of all samples in data-dependent mode on a Q Exactive HF-X orbitrap mass spectrometer (Thermo Fisher Scientific). For data-independent acquisition, each individual sample was injected in at least technical duplicates and measured in data-independent mode with variable isolation window sizes. Spectral library generation as well as DIA analysis was performed in Spectronaut (version 14.3) using the *Q* value sparse setting with a q-value of 0.01 in at least one raw-file using the top3 precursors for quantitation without applying imputation^122^. Further downstream analysis was performed in R. Technical replicates were collapsed into samples by using the average Quantity value. For significance calling on protein level, ratios to the average no-COVID control were calculated, scaled by median-MAD normalization and a one-sample moderated t-test (limma package^123^) was applied among experimental groups. Proteins with a Benjamini-Hochberg adjusted p-value of 0.05 (5% FDR) were considered as significant. Top abundant proteins were subjected to STRING analysis using experiments and databases as interaction sources^124^. For single-sample gene-set enrichment analysis (ssGSEA;^125^), median-MAD scaled log-fold change values of group-wise comparisons were calculated using the mean protein intensity values among each group. Significance calling of pathways was applied on the adjusted p-values from the ssGSEA results. Due to one time-point missing from patient 11, this patient was not included in the proteomic analysis for volcano plots and pathway analysis.

### Interferon plasma assay

For longitudinal assessment of type 1/2/3 interferons plasma levels, plasma samples of control (*n* = 9), non-pneumonic SARS-CoV-2 infected patients (day 4 *n* = 40, day 11 *n* = 18, day 60 *n* = 14) and hospitalized patients suffering from COVID-19 pneumonia (*n* = 7) were analyzed using a commercial multiplex bead-based assay (LEGENDplex™ Human Type 1/2/3 Interferon Panel, V-bottom plate, Biolegend #740396) according to the manufacturer’s instructions. In brief, frozen plasma samples were thawed to room temperature (RT) and centrifuged (1000×*g*, 1 min). Samples were incubated with assay reagents in the dark for 2 h on a plate shaker (RT, 600 rpm). After addition of the detection antibody mix, samples were incubated for another hour (RT, 600 rpm), followed by addition of Streptavidin-PE (30 min, RT, 600 rpm) and an on-plate washing step. Finally, samples were measured in technical duplicates using a BD LSRFortessa flow cytometer. Interferon plasma levels were calculated using LEGENDplex™ Data Analysis Software Suite QOGNIT.

### Statistics and reproducibility

No statistical method was used to predetermine sample size. No data were excluded from the analyses. The experiments were not randomized. The Investigators were not blinded to allocation during experiments and outcome assessment.

### Data analysis and statistics

Excel (Microsoft), Prism (GraphPad) and R with package *corrplot* were used for data analysis. Adobe Illustrator CC was used to assemble the graphical illustrations. Results are shown and mentioned in the text as mean ± s.e.m., unless otherwise indicated. For direct comparisons between two groups, unpaired, two-tailed Student’s *t* tests or Mann–Whitney *U* tests were used. For changes across clusters in flow cytometric measurements mixed-effects model analysis was used. If the mixed-effects model analysis was significant, a line denotes the significance. Post-hoc Sidak’s multiple comparisons test was used for individual significant differences. *P* values of ≤0.05 are considered significant and denoted with *, ≤0.01 with **, and ≤0.001 with ***. Individual patients or samples are represented as dots, unless otherwise indicated. Error bars are standard error of the mean (s.e.m.) unless otherwise indicated.

### Reporting summary

Further information on research design is available in the Nature Research Reporting Summary linked to this article.

## Supplementary information

Supplementary Information

Reporting summary

## Data Availability

RNAseq data generated by this study have been deposited in the Gene Expression Omnibus (GEO) archive with accession number: GSE193708, accessible via. The mass spectrometry proteomics data have been deposited to the ProteomeXchange Consortium (http://proteomecentral.proteomexchange.org) via the PRIDE partner repository with the dataset identifier PXD030785^126^. The processed scRNA-seq data and subset and nasal swab RNA-seq data are available from our Zenodo dataset with 10.5281/zenodo.5792004 (10.5281/zenodo.5792004). Data points in figures are included in the published source data file. All other data is available upon request from the authors. Source data are provided with this paper.

## Source data

Source Data

## Code availability

Custom scripts are available at: https://github.com/mjoppich/covidSC.
