# Supplementary material for: Protective immune trajectories in early viral containment of non-pneumonic SARS-CoV-2 infection
Source: Nat Commun. 2022 Feb 23;13:1018. doi: 10.1038/s41467-022-28508-0 (PMC8866527; doi:10.1038/s41467-022-28508-0)
Supplement: Supplementary file 1 — Supplementary Information [file 41467_2022_28508_MOESM1_ESM.pdf]

Supplementary File

Protective immune trajectories in early viral containment of non-pneumonic SARS-CoV-2 infection

Kami Pekayvaz[1,2,#,*], Alexander Leunig[1,2,#], Rainer Kaiser[1,2], Markus Joppich[3], Sophia Brambs[1], , Aleksandar Janjic[4], Oliver Popp[5], Daniel Nixdorf[6,7], Valeria Fumagalli[8, 9], Nora Schmidt[10], Vivien Polewka[1], Afra Anjum[1], Viktoria Knottenberg[1], Luke Eivers[1], Lucas E. Wange[4], Christoph Gold[1], Marieluise Kirchner[5], Maximilian Muenchhoff[11,12,13], Johannes C. Hellmuth[7,13], Clemens Scherer[1,2,13], Raquel Rubio-Acero[12,14], Tabea Eser[12,14], Flora Deák[12,14], Kerstin Puchinger[14], Niklas Kuhl[15,16], Andreas Linder[15,16], Kathrin Saar[5], Lukas Tomas[1,2], Christian Schulz[1,2], Andreas Wieser[12,14], Wolfgang Enard[4], Inge Kroidl[12,14], Christof Geldmacher[12,14], Michael von Bergwelt-Baildon[7,13], Oliver T. Keppler[11,12,13], Mathias Munschauer[10], Matteo Iannacone[8, 9, 17], Ralf Zimmer[3], Philipp Mertins[5], Norbert Hubner[5], Michael Hoelscher[12,14], Steffen Massberg[1,2], Konstantin Stark[1,2,§,*], Leo Nicolai[1,2,§,*]

Affiliations

1 Medizinische Klinik und Poliklinik I University Hospital Ludwig-Maximilian University Munich, Germany
2 DZHK (German Centre for Cardiovascular Research), partner site Munich Heart Alliance, Germany
3 Department of Informatics, Ludwig-Maximilian Universität München, Germany
4 Anthropology and Human Genomics, Faculty of Biology, Ludwig-Maximilians University Munich
5 Max Delbrück Center for Molecular Medicine (MDC) and Berlin Institute of Health (BIH), Berlin, Germany
6 Laboratory for Translational Cancer Immunology, Gene Center, LMU Munich, Munich, Germany
7 Medizinische Klinik und Poliklinik III, University Hospital Ludwig-Maximilian University Munich, Germany
8 Division of Immunology, Transplantation and Infectious Diseases, IRCCS San Raffaele Scientific Institute, 20132 Milan, Italy
9 Vita-Salute San Raffaele University, 20132 Milan, Italy
10 Helmholtz Institute for RNA-based Infection Research, Helmholtz-Center for Infection Research, Würzburg, Germany
11 Max von Pettenkofer Institute & GeneCenter, Virology, Faculty of Medicine, LMU München, Munich, Germany
12 German Center for Infection Research (DZIF), Partner Site Munich
13 COVID-19 Registry of the LMU Munich (CORKUM), University Hospital, LMU Munich
14 Division of Infectious Diseases and Tropical Medicine, University Hospital Ludwig-Maximilian University Munich, Germany
15 Gene Center and Department of Biochemistry, University Hospital Ludwig-Maximilian University Munich, Germany
16 Medizinische Klinik und Poliklinik II, University Hospital Ludwig-Maximilian University Munich, Germany
17 Experimental Imaging Centre, IRCCS San Raffaele Scientific Institute, 20132 Milan, Italy
# Contributed equally
§ Jointly supervised this work
* Correspondence to: kami.pekayvaz@med.uni-muenchen.de, konstantin.stark@med.uni-muenchen.de or leo.nicolai@med.uni-muenchen.de

Supplementary Figures

Suppl Fig 1

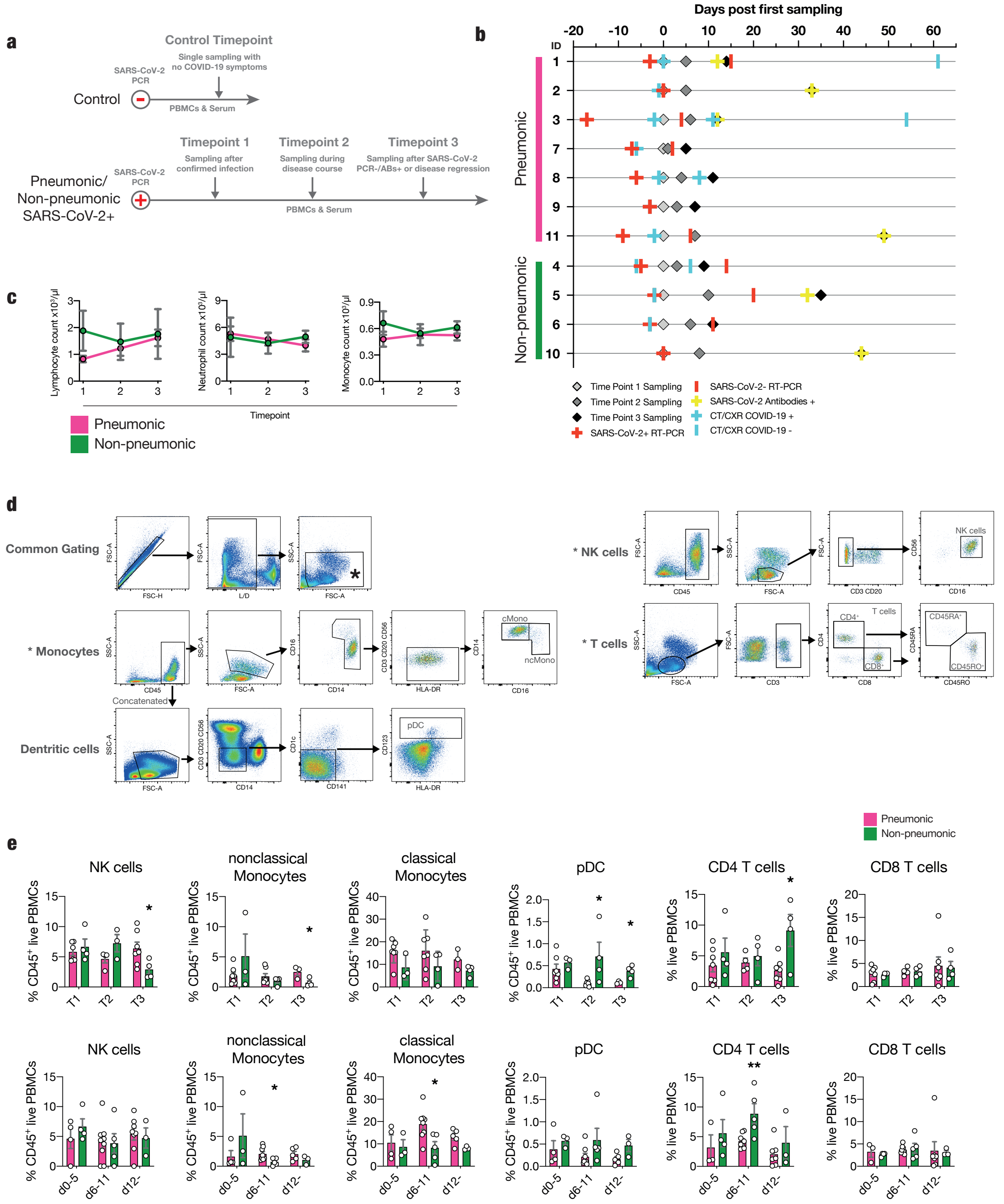

Suppl. Fig. 1 | a: Schematic of exploratory cohort and sampling time points. **b:** Timeline of the exploratory group normalized by the first sampling time point (day=0). Sampling time points, positive and negative real time polymerase chain reaction (RT-PCR) for SARS-CoV-2, positive and negative COVID-19 in chest tomography/chest x-ray and positive SARS-CoV-2 antibody detection are shown for n=7 pneumonic and n=4 non-pneumonic patients. **c:** Longitudinal blood lymphocyte, neutrophil and monocyte counts at time points 1-3 of n=7 pneumonic COVID-19, n≥3 non-pneumonic COVID-19 patients per time point. No statistical testing performed, mean±sem. Exact n numbers per time point are shown in the source data. **d:** Gating strategies for flow-cytometric characterization of PBMCs. Common gating strategy until * is shown in the top row. Further gating strategy is shown for the respective cell population. For dendritic cells, all samples were gated together as a concatenated file. **e:** Percentage of immune cells as live $CD45^+$ cells/PBMCs measured by flow cytometry per sampling time point (top) and binned according to days after SARS-CoV-2PCR+ (bottom). Two-sided t-test, n≥3 per time point and group, mean±sem. Exact n numbers per time point are shown in the source data. *p<0.05, **p<0.01. Source data are provided as a Source Data file.

Suppl Fig 2

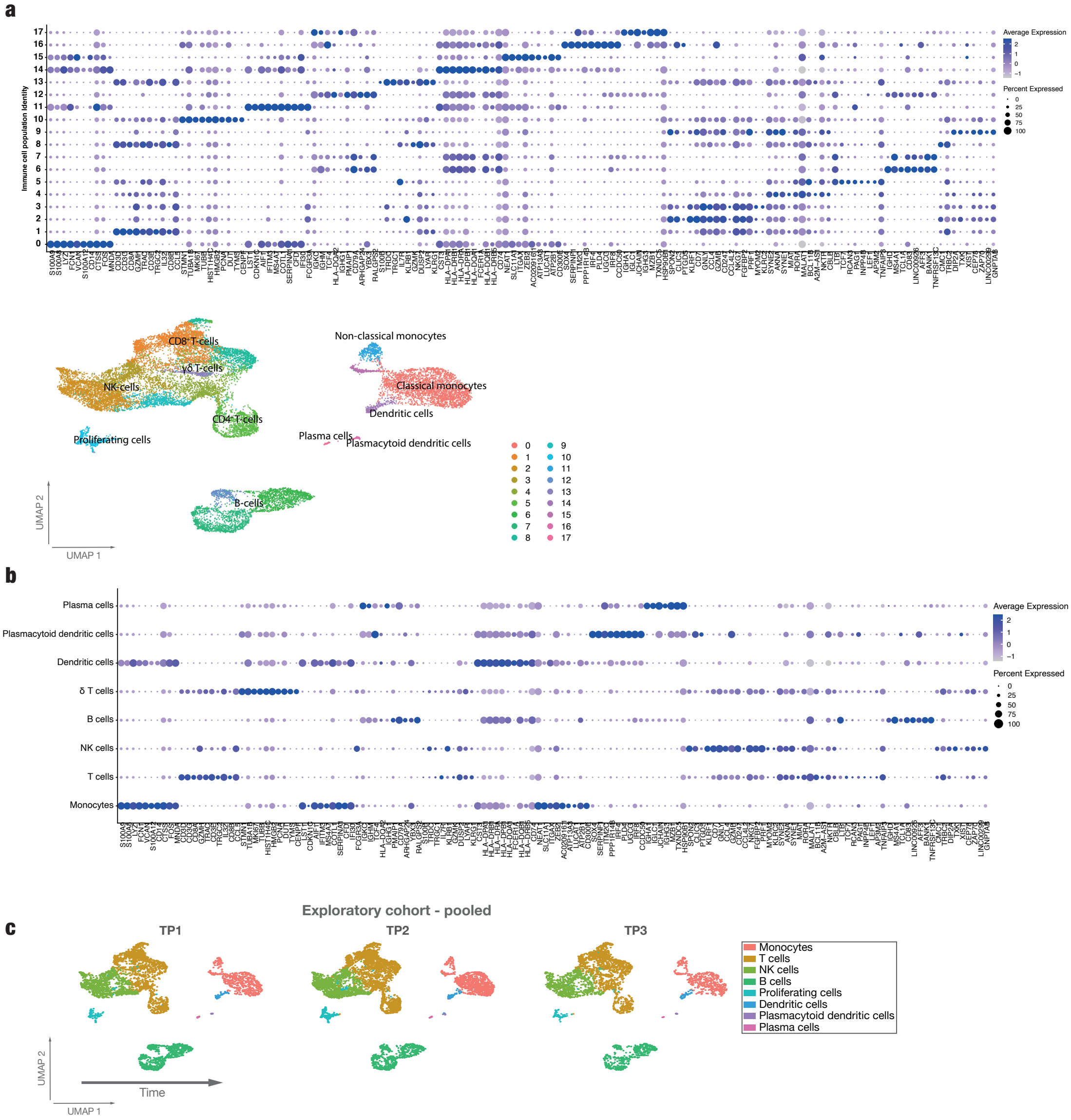

Suppl. Fig. 2 | a: Dot-plot of cluster defining markers for the scRNA-seq data. **b:** Dot-plot of population defining markers for the scRNA-seq data as shown in Figure 1g. **c:** UMAP representation of the exploratory cohort samples showing the assigned cell populations per sampling time point.

Suppl Fig 3

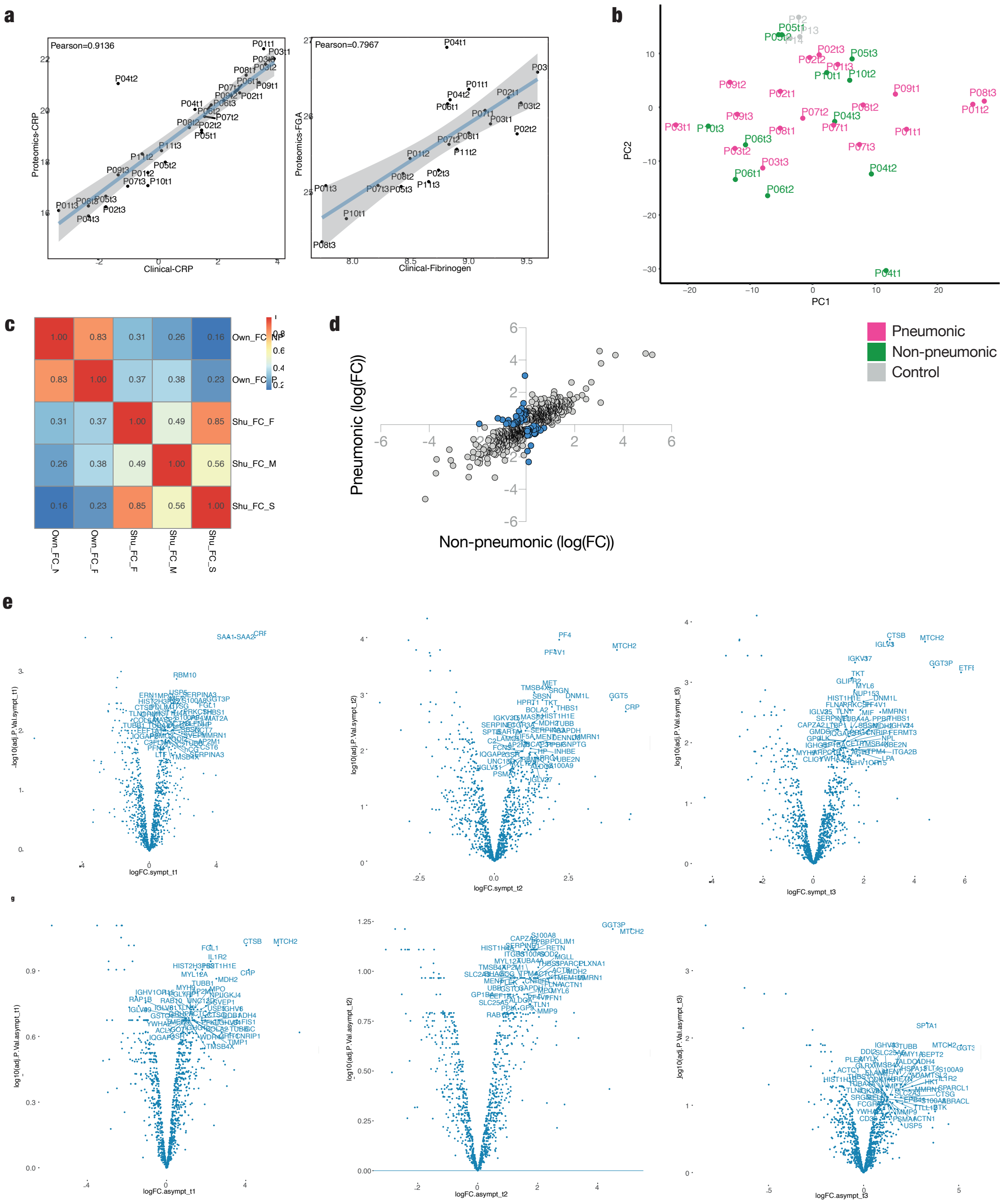

Suppl Fig. 3 | a: Correlation analyses between plasma proteomic measurement and clinical laboratory measurement of CRP and fibrinogen (FGA). Pearson correlation coefficient shown on top. Curves show linear fit with 95% confidence interval. **b:** Principal component analysis (PCA) of analysed proteome samples. **c:** Pearson correlation matrix between this proteomic analysis and the proteome from *Shu et al.* Input values are normalized logFC to healthy control samples. **d:** Significantly (adj p<0.05) differentially expressed proteins in plasma samples pooled across all three time points. Log fold change is computed relative to the control protein expression. **e:** Volcano plot of differentially expressed proteins of pneumonic (upper) and non-pneumonic (lower) samples per time point compared to controls. Top proteins are annotated. n=4 patients with 12 samples for non-pneumonic and n=6 patients with 18 samples for pneumonic and n=3 samples and patients for controls. Statistical tests for volcano plots described in methods.

Suppl Fig 4

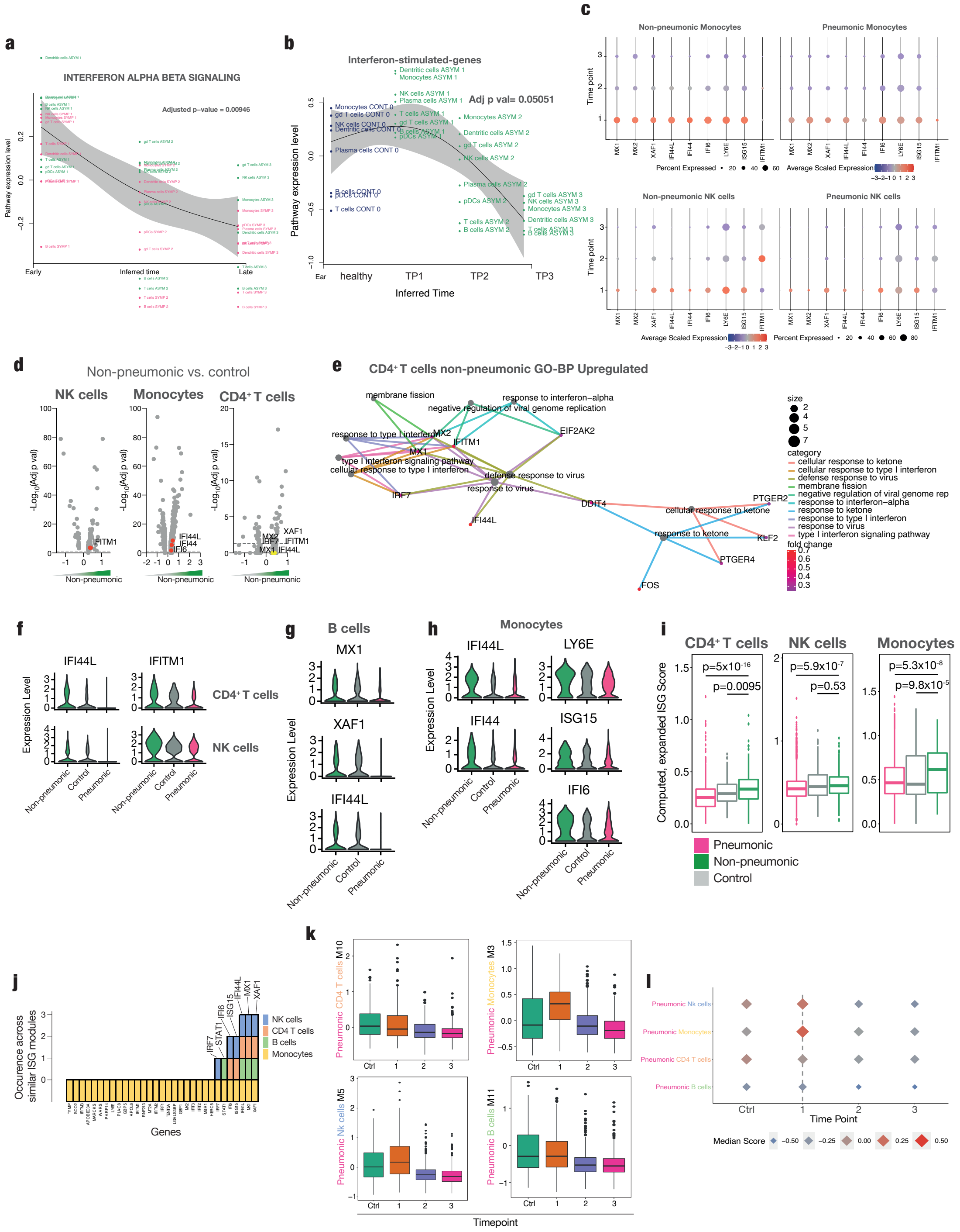

Suppl. Fig. 4 | a: Tempora analysis reveals significant temporal regulation of "Interferon alpha beta signaling" throughout the disease course in non-pneumonic (ASYM) and pneumonic (SYM) subjects, gray area is 95% confidence interval, statistical test conducted with Tempora method. **b:** Longitudinal behavior of predefined ISGs (further depicted in methods) in non-pneumonic patients analysed by Tempora, gray area is 95% confidence interval, statistical test conducted with Tempora method. **c:** Dot-plot of interferon stimulated gene expression and Percent expressing cells in monocytes and NK cells per time point in non-pneumonic and pneumonic patients. **d:** Volcano plot of differentially regulated genes in CD4$^+$ T cells, NK cells and monocytes of non-pneumonic compared to control samples. Red annotations are significantly upregulated (adj p val<0.05). Positive fold change signifies higher expression in the non-pneumonic group. Line denotes adj p val<0.05. Statistical tests for volcano plots described in methods. **e:** GO-BP network analysis of pathways upregulated in CD4$^+$ T cells of non-pneumonic samples compared to pneumonic samples. **f-h:** Violin plots of expression of interferon stimulated in CD4$^+$ T cells, NK cells, C cells and monocytes for pneumonic, non-pneumonic and control samples. **i:** Box plots of expanded ISG-scores (see methods) of CD4$^+$ T cells (n= 725 pneumonic n=388 non- pneumonic and n=162 control cells), NK cells (n= 2550 pneumonic n=888 non- pneumonic and n=903 control cells) and monocytes (n= 1713 pneumonic n=380 non- pneumonic and n=1303 control cells). P-values are shown above, non-pneumonic with pneumonic and control with pneumonic are compared. Box-and-whiskers plot (median, IQR and 1.5IQR), unpaired, two-sided t-tests. **j:** Number of identical occurrence of genes of ISG GMs of pneumonic patients in four immune cell

subsets. **k:** Box-plots showing temporal development of the specific ISG-GMs in pneumonic patients. CD4 T cells n=162,176,347 and 202 per TP respectively, monocytes n=1303,455,796 and 462 per TP respectively, NK cells n=903,749,1038 and 763 per TP respectively and B cells n=440,623,452 and 398 per TP respectively. Box-and-whiskers plot (median, IQR and 1.5IQR). **l:** Dot plots displaying the median score of respective ISG-modules of NK-cells, Monocytes, CD4 T cells, B cells throughout time in pneumonic patients and in control patients. Source data are provided as a Source Data file.

Suppl Fig 5

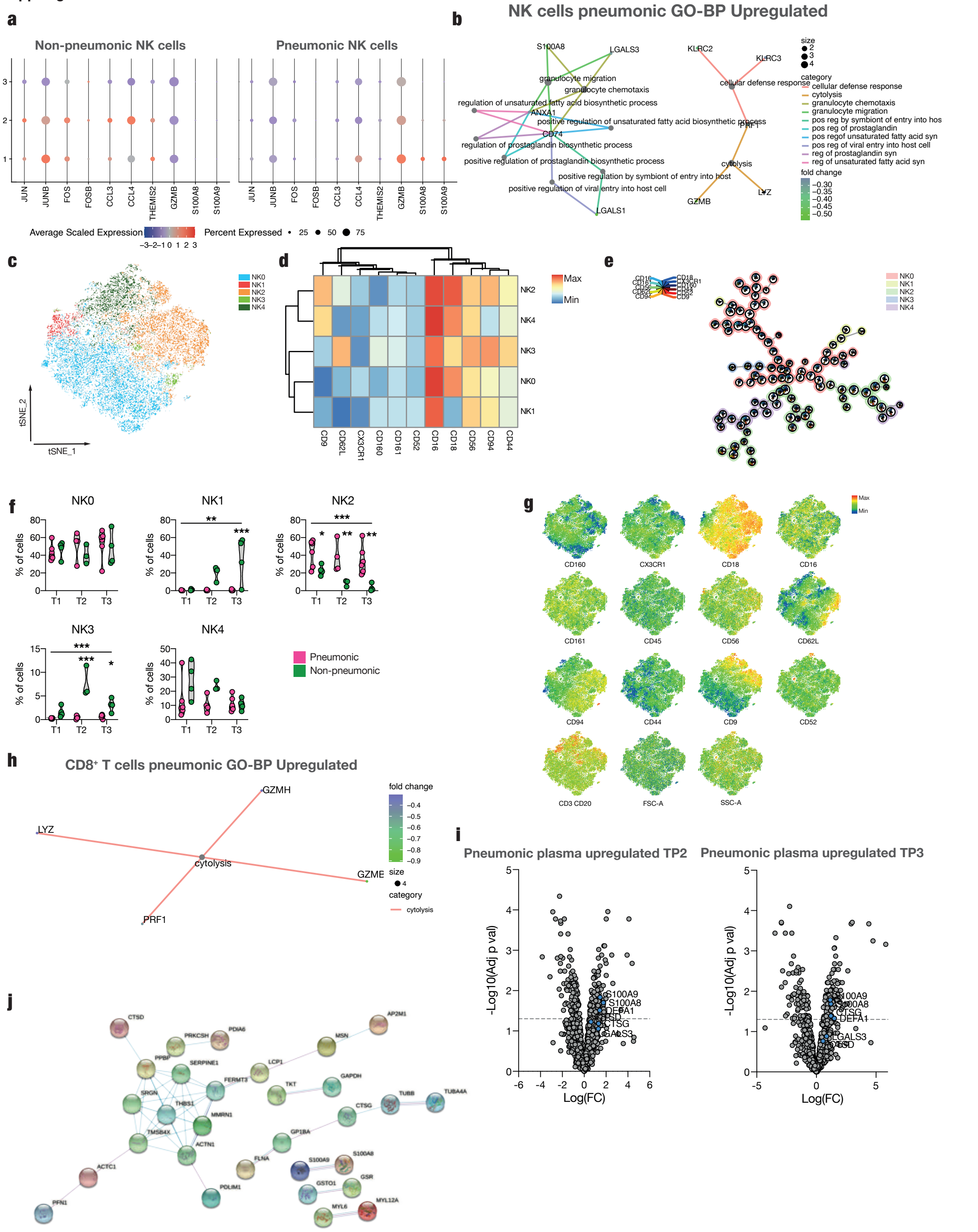

Suppl. Fig. 5 | a: Dot-plot of the scaled average expression and percent expressing cells of selected genes in NK cells by sampling time point in non-pneumonic and pneumonic patients. **b:** GO-BP network analysis of pathways upregulated in NK cells of pneumonic samples compared to non-pneumonic samples. **c:** tSNE of NK cells measured by flow cytometry. FlowSOM assigned clusters are shown in different colors. **d:** Heat map of surface marker expression by FlowSOM group. **e:** FlowSOM self-organizing map of NK cells. **f:** Violin plots of Percentage of NK cells in each FlowSOM cluster per time point. Statistical tests:

Mixed-effects model analysis. Lines denote significant time effect, post-hoc Sidak's multiple comparisons test for individual significant differences between pneumonic and non-pneumonic samples per time point. n≥3 per time point and group. Exact n numbers per time point are shown in the source data. **g:** tSNE with expression for individual surface makers of NK cells. **h:** GO-BP network analysis of pathways enriched in CD8^{+} T cells of pneumonic samples compared to non-pneumonic samples. **i:** Volcano plots of differentially expressed plasma proteins at TP2-3 of pneumonic samples compared to control samples. Line denotes adj p val<0.05. Statistical tests for volcano plots described in methods. **j:** String network of top upregulated proteins in pneumonic plasma samples. *p<0.05, **p<0.01, ***p<0.001. Source data are provided as a Source Data file.

Suppl Fig 6

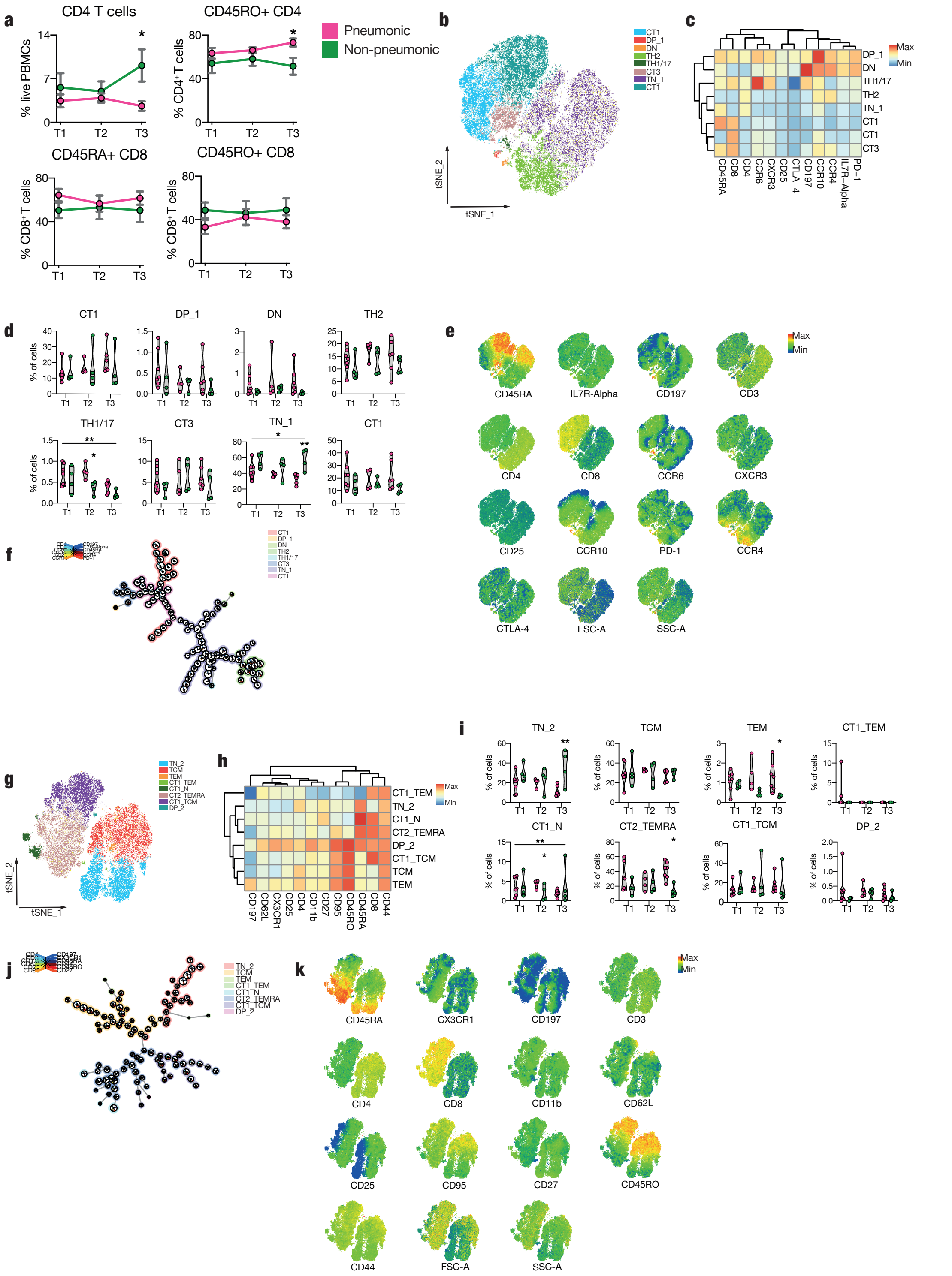

Suppl. Fig. 6 | a: Percentage of CD4^{+} and CD45RA/RO$^{+/-}$ CD8+ / CD4^{+} T cells of live PBMCs measured by flow cytometry per sampling time point. Two-sided t-test, n≥3 per time point and group. Exact n numbers per time point are shown in the source

data. **b:** tSNE of T cells (T cell panel 1) measured by flow cytometry. FlowSOM assigned clusters are shown in different colors. **c:** Heat map of surface marker expression by FlowSOM group. **d:** Violin plots of Percentage of T cells in each FlowSOM cluster per time point. Mixed-effects model analysis. Lines denote significant time effect, post-hoc Sidak's multiple comparisons test for individual significant differences between pneumonic and non-pneumonic samples per time point. n≥4 per time point and group. **e:** tSNE with expression for individual surface makers of T cells. **f:** FlowSOM self-organizing map of T cells. **g:** tSNE of T cells (T cell panel 2) measured by flow cytometry. FlowSOM assigned clusters are shown in different colors. **h:** Heat map of surface marker expression by FlowSOM group. **i:** Violin plots of Percentage of T cells in each FlowSOM cluster per time point. Mixed-effects model analysis. Lines denote significant time effect, post-hoc Sidak's multiple comparisons test for individual significant differences between pneumonic and non-pneumonic samples per time point. n≥4 per time point and group. Exact n numbers per time point are shown in the source data. **j:** FlowSOM self-organizing map of T cells (T cell panel 2). **k:** tSNE with expression for individual surface makers of T cells (T cell panel 2). All error bars are mean ± s.e.m. unless otherwise noted. *p<0.05, **p<0.01. Source data are provided as a Source Data file.

Suppl Fig 7

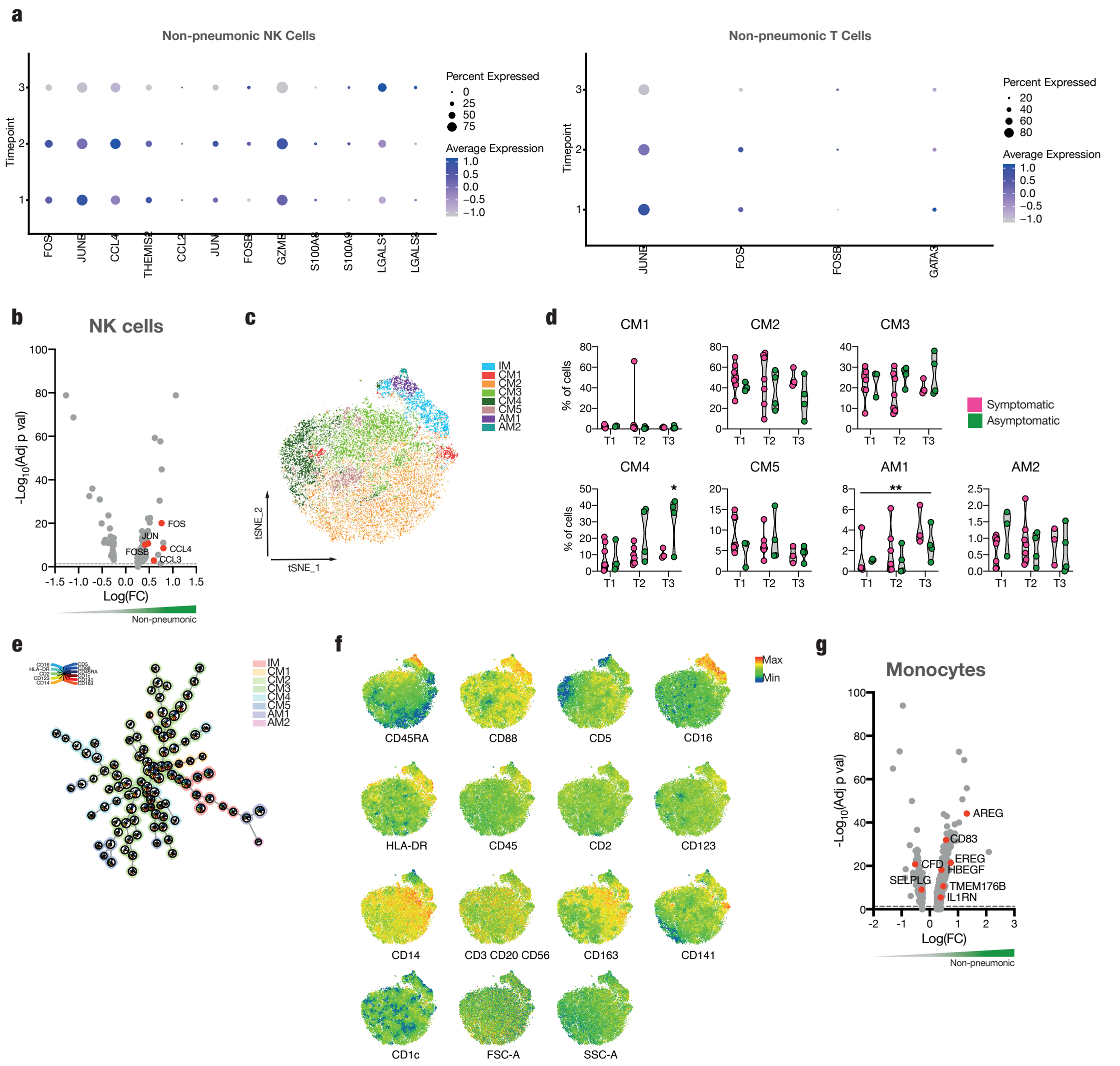

Suppl. Fig. 7 | a: Dot-plot of selected gene expression and percent expressing cells in non-pneumonic and pneumonic NK and T cells per time point. **b:** Volcano plot of differentially up- and downregulated genes in NK cells of non-pneumonic compared to control samples. Red annotations are significantly upregulated adj p val<0.05. Positive fold change signifies higher expression in the non-pneumonic group. Line denotes adj p val<0.05. **c:** tSNE of monocytes measured by flow cytometry. FlowSOM assigned clusters are shown in different colors. **d:** Violin plots of Percentage of monocytes in each FlowSOM cluster per time point. Mixed-effects model analysis. Lines denote significant time effect, post-hoc Sidak's multiple comparisons test for individual significant differences between pneumonic and non-pneumonic samples per time point. n≥3 per time point and group. Exact n numbers per time point are shown in the source data. **e:** FlowSOM self-organizing map of monocytes **f:** tSNE with expression for individual surface makers of monocytes. **g:** Volcano plot of differentially up- and downregulated genes in monocytes of non-pneumonic compared to control samples. Red annotations are significantly upregulated adj p val<0.05. Positive fold change signifies higher expression in the non-pneumonic group. Line denotes adj p val<0.05. Statistical tests for volcano plots described in methods. *p<0.05, **p<0.01. Source data are provided as a Source Data file.

Suppl Fig 8

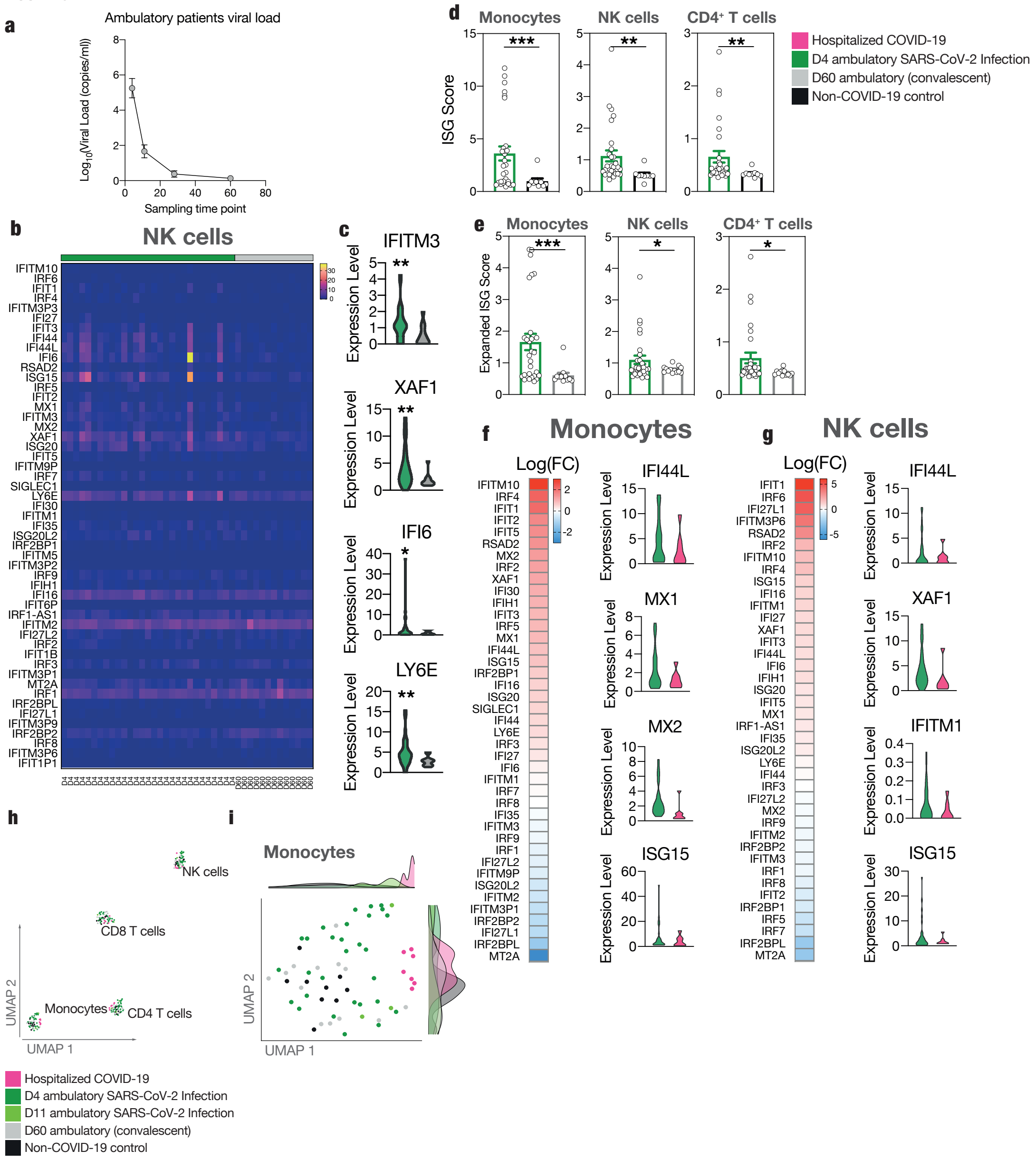

Suppl. Fig. 8 | a: Longitudinal viral load course of n=29 ambulatory patients that were used for leukocyte subset RNA-sequencing. Log_{10}(Viral Load (copies/ml) is depicted at sampling time points day 4, 11, 28 and 60 post positive SARS-CoV-2 PCR. Mean±sem. **b:** Heat map of differentially expressed interferon stimulated genes in NK cells of day 4 ambulatory compared to day 60 (convalescent) SARS-CoV-2 infection. **c:** Individual ISG expressions of exemplary ISGs. Unpaired two-sided t-test with Welch's correction. n=29 d4, n=13 d60. **d:** Computed ISG scores for monocytes, NK cells and $CD4^+$ T cells. **e:** Computed, expanded ISG scores (see methods) for monocytes, NK cells and $CD4^+$ T cells. **d-e:** Unpaired two-sided t-test with Welch's correction. n=29 d4, n=9 non-COVID-19 controls. **f-g:** Heat maps of differentially expressed interferon stimulated genes in leukocyte subsets (monocytes and NK cells) of day 4 ambulatory compared to severe hospitalized COVID-19. Monocytes: n=28 upregulated, n=16 downregulated, NK cells: n=25 upregulated, n=15 downregulated. Individual ISG expressions of exemplary ISGs shown. n=29 d4, n=7 hospitalized. **h:** UMAP depicting single RNA-Seq samples and differentiating different disease severities / timepoints by color. **i:** UMAP further depicting single RNA-Seq monocyte samples, differentiating disease severities / timepoints by color. Mean ± sem is shown unless otherwise specified. *p<0.05, **p<0.01, *** p<0.001. Source data are provided as a Source Data file.

Suppl Fig 9

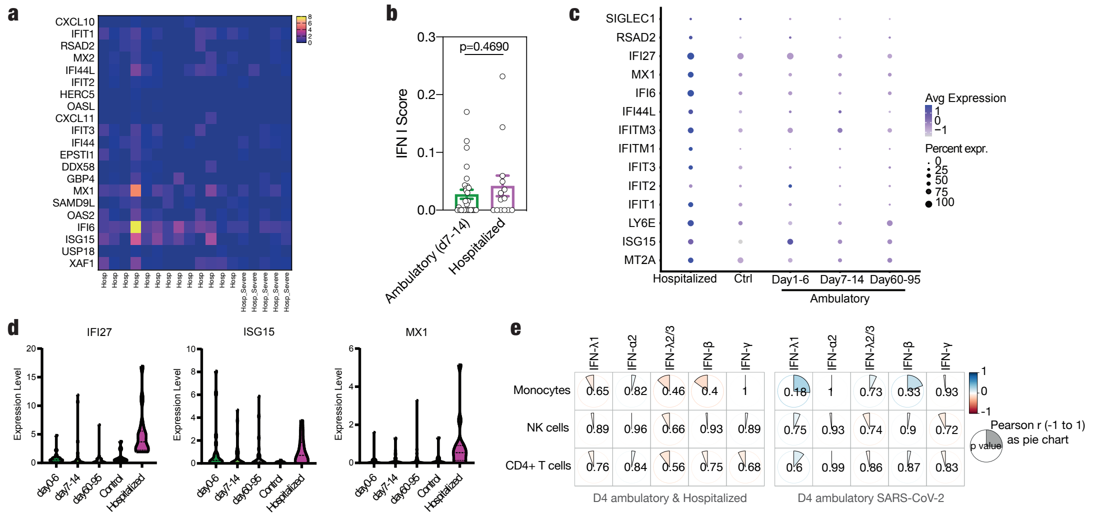

Suppl. Fig. 9 | a: Heat map of nasal swab interferon stimulated genes of the ISG score in Fig **7b**. (n=13 hospitalized, n=5 severe hospitalized non-ICU patients, see methods). **b:** IFN-I score in ambulatory and hospitalized patients. Unpaired two-sided t-test with Welch's correction. **c:** Dot-plot of ISGs across nasal swabs of hospitalized and ambulatory patients and control subjects **d:** Individual ISG expressions of exemplary ISGs, d0-6 n=20, d7-14 n=29, d60-d95 n=28, controls n=10, hospitalized n=14. **e:** Pearson correlation between the five measured interferon levels and ISG scores of monocytes, NK cells and CD4^{+} T of day 4 ambulatory COVID-19 patients and hospitalized COVID-19 patients (pooled and day 4 of ambulatory patients alone). P value is shown in center of each field (none <0.05). Pie charts show Pearson's r from maximum 1 (clockwise and blue) to -1 (anticlockwise and red). n=27 day 4 and n=7 hospitalized COVID-19. Mean ± sem is shown unless otherwise specified. Source data are provided as a Source Data file.

Supplementary Tables

Supplementary Table 1: WHO grade equivalent of cohorts

Cohort	Group	Description	Equivalent WHO Grade
Exploratory Cohort	Non-pneumonic	SARS-CoV-2+ patients without any pulmonary symptoms or radiological infiltrates.	3-4
Exploratory Cohort	Pneumonic	SARS-CoV-2+ patients with confirmed COVID-19 associated pneumonia	3-4
Exploratory Cohort	Control	SARS-CoV-2- patients	0
Confirmation Cohort	Ambulatory d0-14	Ambulatory SARS-CoV-2+ patients	1-2
Confirmation Cohort	Hospitalized (without subdifferentiation)	SARS-CoV-2+ patients on a general hospital ward without major symptoms	3-4
Confirmation Cohort	Hospitalized_mild	SARS-CoV-2+ patients on a general hospital ward without major symptoms	3-4
Confirmation Cohort	Hospitalized_severe	SARS-CoV-2+ hospitalized patients with severe disease (see methods)	4-5
Confirmation Cohort	Ambulatory d60-95	Ambulatory SARS-CoV-2+ patients after reconvalescence	0
Confirmation Cohort	Control	Ambulatory control patients (healthy individuals)	0

Table 2: Viral loads of cohorts

Cohort	Group	Timepoint/batch	$\log_{10}$(viral load) Median [IQR]
Confirmation cohort	Hospitalized	d5	5.7 [IQR: 3.1, 7.0]
Confirmation cohort	Ambulatory	d4	5.6 [IQR: 3.1, 7.0]
Confirmation cohort	Ambulatory	d11	1.0 [IQR: 0, 3.3]
Confirmation cohort	Ambulatory	d28	0.0 [IQR: 0.0, 0.0]
Confirmation cohort	Ambulatory	d60	0.0 [IQR: 0.0, 0.0]
Nasal swabs	Hospitalized	All (day7-14)	4.8 [IQR: 3.8, 6.5]
Nasal swabs	Hospitalized	severe only	6.1 [IQR: 4.5, 6.3]
Nasal swabs	Hospitalized	normal only	4.1 [IQR: 3.8, 7.1]
Nasal swabs	Ambulatory	day 0-6	8.3 [IQR: 5.3, 8.8]
Nasal swabs	Ambulatory	day 7-14	4.7 [IQR: 3.3, 6.5]
Nasal swabs	Ambulatory	day 60-95	0.0 [IQR: 0.0, 0.0]
Exploratory cohort	Pneumonic	Viral Load TP1	4.3 [IQR: 3.8, 5.3]
Exploratory cohort	Pneumonic	Viral Load TP2	3.0 [IQR: 2.8, 3.8]
Exploratory cohort	Pneumonic	Viral Load TP3	2.7 [IQR: 1.8, 3.2]
Exploratory cohort	Non-pneumonic	Viral Load TP1	5.2 [IQR: 3.0, 7.6]
Exploratory cohort	Non-pneumonic	Viral Load TP2	3.7 [IQR: 3.0, 5.1]
Exploratory cohort	Non-pneumonic	Viral Load TP3	2.7 [IQR: 1.3, 3.0]

Supplementary Table 3: Monocyte Panel – subset descriptions

Description	Name
Intermediate Monocyte	IM
Classical Monocyte	CM1
Classical Monocyte	CM2
Classical Monocyte	CM3
Classical Monocyte	CM4
Classical Monocyte	CM5
Alternative Monocyte	AM1
Alternative Monocyte	AM2

Supplementary Table 4: T cell panel 1 – subset descriptions

Description	Name
Cytotoxic CD8 TC	CT1
double positive TC	DP_1
double negative TC	DN
TH2 CD4 TC	TH2
TH1/17 CD4 TC	TH1/17
naive cytotoxic CD8 TC	CT3
naive CD4 TC	TN_1
cytotoxic CD8 TC	CT1

Supplementary Table 5: T Cell Panel 2 – subset descriptions

Description	Name
naive CD4 TC	TN_2
central memory CD4 TC	TCM
effector memory CD4 TC	TEM
cytotoxic effector memory CD8 TC	CT1_TEM
cytotoxic naive CD8 TC	CT1_N
cytotoxic TEMRA CD8 TC	CT2_TEMRA
cytotoxic central memory CD8 TC	CT1_TCM
double positive TC	DP_2

Supplementary Table 6: NK Cell Panel - Antibodies used

Color	Antigen	Company & Cat #
BV650	CD45	BioLegend #304044
FITC	CD94	BioLegend #305504
PE	CD3	BioLegend #300308
PE	CD20	BioLegend #302306
PERCP-Cy5.5	CD44 (RAT)	BioLegend #103032

APC	CD160	BioLegend #341208
APC-Cy7	CX3CR1 (RAT)	BioLegend #341616
BV 605	CD161	BioLegend #339916
BV785	CD62L	BioLegend #304830
BV510	CD16	BioLegend #302048
BV 711	CD56	BioLegend #362542
AF 700	CD18	BioLegend #302124
PE-Dazzle	CD52	BioLegend #316014
PE-Cy7	CD9	BioLegend #312116

Supplementary Table 7: Monocyte Panel - Antibodies used

Color	Antigen	Company & Cat #
BV650	CD45	BioLegend #304044
FITC	CD14	BD Biosciences #557153
PE	CD3	BioLegend #300308
PE	CD20	BioLegend #302306
PE	CD56	BioLegend #304606
PERCP-Cy5.5	CD1c	BioLegend #331514
APC	CD45RA	BioLegend #304112
APC-Cy7	CD88	BioLegend #344316
BV 605	HLA-DR	BioLegend #307640
BV785	CD123	BioLegend #306032
BV510	CD16	BioLegend #302048
BV 711	CD2	BioLegend #300232
AF 700	CD5	BioLegend #364026

Supplementary Table 8: T Cell Panel 1- Antibodies used

Color	Antigen	Company & Cat #
BV650	CD8	BioLegend #344730
FITC	CD25	BioLegend #302604
PE	CCR10	BioLegend #341504
PERCP-Cy5.5	CD152 (CTLA-4)	BioLegend #369608
APC	CD45RA	BioLegend #304112
APC-Cy7	CD127 (IL7Ra)	BioLegend #135040
BV605	CD4	BioLegend #317438
BV785	CXCR3	BioLegend #353738
BV510	CD3	BioLegend #317332
BV711	CD196 (CCR6)	BioLegend #353436
AF700	CD197	BioLegend #353244
PE-Dazzle	CCR4	BioLegend #359420
PE-Cy7	CD279 (PD-1)	BioLegend #621616

Supplementary Table 9: T Cell Panel 2- Antibodies used

Color	Antigen	Company & Cat #
BV650	CD8	BioLegend #344730
FITC	CD25	BioLegend #302604

PE	CD95	BioLegend #305608
PERCP-Cy5.5	CD44 (RAT)	BioLegend #103032
APC	CD45RA	BioLegend #304112
APC-Cy7	CX3CR1 (RAT)	BioLegend #341616
BV 605	CD4	BioLegend #317438
BV785	CD62L	BioLegend #304830
BV510	CD3	BioLegend #317332
BV 711	CD11b	BioLegend #301344
AF 700	CD197	BioLegend #353244
PE-Dazzle	CD45RO	BioLegend #304248
PE-Cy7	CD27	BioLegend #356412
