# Supplementary material for: Protective immune trajectories in early viral containment of non-pneumonic SARS-CoV-2 infection
Source: Nat Commun. 2022 Feb 23;13:1018. doi: 10.1038/s41467-022-28508-0 (PMC8866527; doi:10.1038/s41467-022-28508-0)
Supplement: Supplementary file 2 — Reporting summary [file 41467_2022_28508_MOESM2_ESM.pdf]

nature research

Corresponding author(s): Kami Pekayvaz, Konstantin Stark, Leo Nicolai

Last updated by author(s): Jan 15, 2022

Reporting Summary

Nature Research wishes to improve the reproducibility of the work that we publish. This form provides structure for consistency and transparency in reporting. For further information on Nature Research policies, see our Editorial Policies and the Editorial Policy Checklist.

Statistics

For all statistical analyses, confirm that the following items are present in the figure legend, table legend, main text, or Methods section.

n/a	Confirmed	
☐	☒	The exact sample size (n) for each experimental group/condition, given as a discrete number and unit of measurement
☐	☒	A statement on whether measurements were taken from distinct samples or whether the same sample was measured repeatedly
☐	☒	The statistical test(s) used AND whether they are one- or two-sided<br>*Only common tests should be described solely by name; describe more complex techniques in the Methods section.*
☐	☒	A description of all covariates tested
☐	☒	A description of any assumptions or corrections, such as tests of normality and adjustment for multiple comparisons
☐	☒	A full description of the statistical parameters including central tendency (e.g. means) or other basic estimates (e.g. regression coefficient) AND variation (e.g. standard deviation) or associated estimates of uncertainty (e.g. confidence intervals)
☐	☒	For null hypothesis testing, the test statistic (e.g. F, t, r) with confidence intervals, effect sizes, degrees of freedom and P value noted<br>*Give P values as exact values whenever suitable.*
☒	☐	For Bayesian analysis, information on the choice of priors and Markov chain Monte Carlo settings
☒	☐	For hierarchical and complex designs, identification of the appropriate level for tests and full reporting of outcomes
☐	☒	Estimates of effect sizes (e.g. Cohen's d, Pearson's r), indicating how they were calculated

Our web collection on statistics for biologists contains articles on many of the points above.

Software and code

Policy information about availability of computer code

Data collection	BD FACSDiva v9.0<br>BD FACSSymphony A5 SORP

Data analysis	fastqc (version 0.11.)<br>Cutadapt (version 1.12)<br>zUMIs pipeline (version 2.9.4d)<br>Human GRCh38 reference genome with Gencode annotation (v35)<br>STAR (version 2.7.3a)<br>RSubread (version 1.32.4)<br>FlowJo Software (FlowJo v. 10.6.1, BD), FlowSOM v2.5 plugin<br>Spectronaut (version 14.3)<br>Microsoft Excel (2019)<br>Prism 9 (GraphPad)<br>cellranger 4.0.0<br>R (version 4.0.3): Seurat (3.2.1 and 4.0.2), clusterProfiler (3.18.1), Tempora (0.1.0), DESeq2 (1.30), limma (3.46.0), WGCNA (1.69), EdgeR (3.32.1)<br>RNA-seq analysis scripts are available from https://github.com/mjoppich/covidSC<br>cPred with PanglaoDB marker genes (https://github.com/mjoppich/scrnaseq_celltype_prediction)<br>Adobe illustrator CC<br>FlowSOM v2.5

For manuscripts utilizing custom algorithms or software that are central to the research but not yet described in published literature, software must be made available to editors and reviewers. We strongly encourage code deposition in a community repository (e.g. GitHub). See the Nature Research guidelines for submitting code & software for further information.

Data

Policy information about availability of data

All manuscripts must include a data availability statement. This statement should provide the following information, where applicable:

- Accession codes, unique identifiers, or web links for publicly available datasets
- A list of figures that have associated raw data
- A description of any restrictions on data availability

Raw data from in vitro experiments will be provided in Table format. Seq data analysis is available at https://github.com/mjoppich/covidSC. The data upload links have been included in the manuscript. We will freely share all data upon reasonable request.

Field-specific reporting

Please select the one below that is the best fit for your research. If you are not sure, read the appropriate sections before making your selection.

- [x] Life sciences
- [ ] Behavioural & social sciences
- [ ] Ecological, evolutionary & environmental sciences

For a reference copy of the document with all sections, see nature.com/documents/nr-reporting-summary-flat.pdf

Life sciences study design

All studies must disclose on these points even when the disclosure is negative.

Sample size	Sample sizes were selected by studying comparable studies published and taking into account local ressources as well as sample availability.
Data exclusions	No data exclusion was performed.
Replication	We successfully replicated our key findings generated in scRNA seq data with comparably small sample sizes in a larger, completely independent cohort using different technology and deeper sequencing. Figure legends contain all details on replicate experiments and sample sizes for presented data, and all attempts at replication were successful.
Randomization	As this was a descriptive study without intervention, no randomization was performed or necessary. Matched pairs were selected in cohort 1 from the CORKUM registry accoring to disease presentation. In cohort 2, all available patients were included.
Blinding	No blinding was performed as the complexity of data did require allocation of samples to respective clinical groups (e.g. pneumonic and non-pneumonic).

Reporting for specific materials, systems and methods

We require information from authors about some types of materials, experimental systems and methods used in many studies. Here, indicate whether each material, system or method listed is relevant to your study. If you are not sure if a list item applies to your research, read the appropriate section before selecting a response.

Materials & experimental systems

n/a	Involved in the study	
[ ]	[x]	Antibodies
[x]	[ ]	Eukaryotic cell lines
[x]	[ ]	Palaeontology and archaeology
[x]	[ ]	Animals and other organisms
[ ]	[x]	Human research participants
[ ]	[x]	Clinical data
[x]	[ ]	Dual use research of concern

Methods

n/a	Involved in the study	
[x]	[ ]	ChIP-seq
[ ]	[x]	Flow cytometry
[x]	[ ]	MRI-based neuroimaging

Antibodies

Antibodies used

BV650 CD45 BioLegend #304044
FITC CD94 BioLegend #305504
PE CD3 BioLegend #300308
PE CD20 BioLegend #302306
PERCP-Cy5.5 CD44 (RAT) BioLegend #103032
APC CD160 BioLegend #341208
APC-Cy7 CX3CR1 (RAT) BioLegend #341616
BV 605 CD161 BioLegend #339916
BV785 CD62L BioLegend #304830
BV510 CD16 BioLegend #302048
BV 711 CD56 BioLegend #362542
AF 700 CD18 BioLegend #302124
PE-Dazzle CD52 BioLegend #316014
PE-Cy7 CD9 BioLegend #312116
BV650 CD45 BioLegend #304044
FITC CD14 BD Biosciences #557153
PE CD3 BioLegend #300308
PE CD20 BioLegend #302306
PE CD56 BioLegend #304606
PERCP-Cy5.5 CD1c BioLegend #331514
APC CD45RA BioLegend #304112
APC-Cy7 CD88 BioLegend #344316
BV 605 HLA-DR BioLegend #307640
BV785 CD123 BioLegend #306032
BV510 CD16 BioLegend #302048
BV 711 CD2 BioLegend #300232
AF 700 CD5 BioLegend #364026
BV650 CD8 BioLegend #344730
FITC CD25 BioLegend #302604
PE CCR10 BioLegend #341504
PERCP-Cy5.5 CD152 (CTLA-4) BioLegend #369608
APC CD45RA BioLegend #304112
APC-Cy7 CD127 (IL7Ra) BioLegend #135040
BV605 CD4 BioLegend #317438
BV785 CXCR3 BioLegend #353738
BV510 CD3 BioLegend #317332
BV711 CD196 (CCR6) BioLegend #353436
AF700 CD197 BioLegend #353244
PE-Dazzle CCR4 BioLegend #359420
PE-Cy7 CD279 (PD-1) BioLegend #621616
BV650 CD8 BioLegend #344730
FITC CD25 BioLegend #302604
PE CD95 BioLegend #305608
PERCP-Cy5.5 CD44 (RAT) BioLegend #103032
APC CD45RA BioLegend #304112
APC-Cy7 CX3CR1 (RAT) BioLegend #341616
BV 605 CD4 BioLegend #317438
BV785 CD62L BioLegend #304830
BV510 CD3 BioLegend #317332
BV 711 CD11b BioLegend #301344
AF 700 CD197 BioLegend #353244
PE-Dazzle CD45RO BioLegend #304248

PE-Cy7 CD27 BioLegend #356412
PE CD3 BioLegend #300308
PE CD20 BioLegend #302306
FITC CD14 BD Biosciences #557153
PE-Cy7 CD16 BD Biosciences #557744
APC-Cy7 CD56 BioLegend #362512
PE-Cy7 CD4 BioLegend #357410
AF488 CD8a BioLegend #301024
APC-Cy7 CD19 BioLegend #363010

Validation

All antibodies used are commercially available and tested products. We performed internal validation of antibodies. Bead based compensation was performed.

Human research participants

Policy information about studies involving human research participants

Population characteristics

Exploratory cohort: For our exploratory cohort, a subset of patients without any pulmonary involvement were included as a model immune response to SARS-CoV-2. In total, 14 subjects were included in our exploratory cohort (n=11 patients with positive SARS-CoV-2 RT-PCR and n=3 non-COVID-19 control subjects). Patients with severe pre-existing kidney or liver dysfunction, severe autoimmune diseases, chronic infection, patients requiring ECMO therapy, with a known coinfection with Influenza or Respiratory Syncytial Virus (RSV) were excluded. COVID-19 patients were divided into patients without any pulmonary symptoms or radiological infiltrates and patients with confirmed COVID-19 associated pneumonia. Furthermore, control subjects without COVID-19 were included. Average number of risk factors was calculated based on individual risk factor sum. Risk factors were medical or physiological conditions associated with severe COVID-19: Age>60yrs, arterial hypertension, cardiovascular disease, chronic respiratory disease, diabetes mellitus, and male gender.
Confirmation cohort: The confirmation cohort consisted of a total of 58 patients. Of these, n=42 were SARS-CoV-2 positive, non-hospitalized individuals. These 42 patients participated in the longitudinal KoCo19-Immu cohort, which enrolled SARS CoV-2 infected individuals shortly after PCR confirmation. Comprehensive longitudinal blood sampling was performed by household visits of field teams. A subset of n=40 longitudinally sampled, ambulatory patients were used for plasma cytokine analysis, with n=40 d4, n=18 d11, n=14 d60 samples measured. Another subset of n=39 ambulatory patients were used for subset RNA sequencing, with n=39 d4, n=13 d60 samples used. In addition to these 42 ambulatory patients, a reference cohort of SARS-CoV-2 (n=9) negative individuals (female: 78%, median age: 27) and hospitalized COVID-19 (n=7) were recruited (female: 29%, median age: 82). The confirmation cohort was sampled and processed completely independently from the exploratory cohort to reduce any systemic bias. The KoCo19-Immu-study is conducted under the framework of the prospective population based Koco19 cohort.

Recruitment

Patients were included in the two cohorts by physicians of the university hospital during hospital stay and/or in the ambulatory testing setting. No obvious bias was discernable.

Ethics oversight

In accordance with the Declaration of Helsinki, and with the approval of the Ethics Committee of Ludwig-Maximilian-University Munich, informed consent of the patients or their guardians was obtained. COVID-19 patients are part of the COVID-19 Registry of the LMU University Hospital Munich (CORKUM, WHO trial ID DRKS00021225). Pseudonymized data was used for analysis, the CORKUM and KocoImmu studies were approved by the ethics committee of LMU Munich (No: 20-245 & No: 20-371 respectively).

Note that full information on the approval of the study protocol must also be provided in the manuscript.

Clinical data

Policy information about clinical studies
All manuscripts should comply with the ICMJEguidelines for publication of clinical research and a completedCONSORT checklist must be included with all submissions.

Clinical trial registration

WHO trial ID DRKS00021225

Study protocol

The full protocol can be seen at lmu-klinikum.de

Data collection

We analyzed n=97 PBMC samples, n=124 plasma samples and n=105 swabs from a total of 104 individual patients.
Two independent cohorts were used for PBMC/plasma analyses, an exploratory cohort (scRNA-Seq, flow cytometry, plasma proteomics) and a confirmation cohort (leukocyte subset in-depth RNA-Seq, cytokine assay). The exploratory cohort was included for hypothesis generation. This was subsequently validated by the confirmation cohort, consisting of PBMC/plasma analyses from independent patients.
In addition, we included nasal swab analyses from both hospitalized and ambulatory patients that were either already included in the two independent cohorts mentioned above (n=37) or were additionally recruited (n=32).
Exploratory cohort: In total, 14 subjects were included in our exploratory cohort (n=11 patients with positive SARS-CoV-2 RT-PCR and n=3 non-COVID-19 control subjects). COVID-19 patients were sampled longitudinally, and three time points were included: median day 3.0 [IQR 2.5,6.5] first sampling, median day 8.0 [IQR 8,11] second sampling and median day 17 [IQR 14,35] third sampling. 14 patients were included for flow cytometric analysis, 12 patients were included into single cell RNA-Seq assays. Patients with severe pre-existing kidney or liver dysfunction, severe autoimmune diseases, chronic infection, patients requiring ECMO therapy, with a known coinfection with Influenza or Respiratory Syncytial Virus (RSV) were excluded. COVID-19 patients were divided into patients without any pulmonary symptoms or radiological infiltrates and patients with confirmed COVID-19 associated pneumonia.

Furthermore, control subjects without COVID-19 were included. Average number of risk factors was calculated based on individual risk factor sum. Risk factors were medical or physiological conditions associated with severe COVID-19: Age>60yrs, arterial hypertension, cardiovascular disease, chronic respiratory disease, diabetes mellitus, and male gender.

Confirmation cohort: The confirmation cohort consisted of a total of 58 subjects included for circulating blood leukocyte subset and cytokine assays. Of these, n=42 were SARS-CoV-2 positive, non-hospitalized individuals. These patients participated in the longitudinal KoCo19-Immu cohort, which enrolled SARS CoV-2 infected individuals shortly after PCR confirmation. Comprehensive longitudinal blood sampling as well as nasopharyngeal swabs were performed by household visits of field teams. Additionally, PBMC samples from n= 7 hospitalized COVID-19 patients on normal wards were used, as well as PBMC samples from n=9 control patients without COVID-19.

For leukocyte subset RNA Seq and cytokine profiling, the ambulatory SARS CoV-2 infected individuals were analyzed at three time points after initial RT-PCR confirmed COVID-19 infection: first at day 4 after RT-PCR, second at day 11 and third at day 60 after positive RT-PCR. The median day for the first timepoint was on day 6 after symptom onset [IQR: 5 to 9.75 days], the median for the second was on day 15 [IQR: 11.75 – 17.25] and for the last visit on day 68 [IQR: 63.25 – 83.25] after symptom onset. The hospitalized COVID-19 patients in this sub-cohort were analyzed at median day 5 after positive RT-PCR or symptom onset [IQR: 3.0 – 15.0]. A subset of n=40 longitudinally sampled, ambulatory patients were used for plasma cytokine analysis, with n=40 d4, n=18 d11, n=14 d60 samples measured. Another subset of n=39 ambulatory patients were used for subset RNA sequencing, with n=39 d4, n=13 d60 samples used. In addition to these 42 total ambulatory patients, a reference cohort of SARS-CoV-2 (n=9) negative individuals (female: 78%, median age: 27) and hospitalized COVID-19 (n=7) were recruited for the subset RNA seq and cytokine assays (female: 29%, median age: 82). The confirmation cohort was sampled and processed completely independently from the exploratory cohort to reduce any systemic bias. The KoCo19-Immu-study is conducted under the framework of the prospective population based Koco19 cohort 72, 73. The upper respiratory tract viral load of the ambulatory cohort at day 4 was median 5.3 [IQR: 3.2, 7.0] log10(Viral load (copies/ml)), and of hospitalized patients median 5.7 [IQR: 3.1, 7.0]. A viral load time course of the ambulatory patients is depicted in Supplementary Figure 8a.

Nasal swab samples: For nasal swab analysis the cohort included a total of 69 patients. Nasal swabs were included from both hospitalized and ambulatory patients that were either already included in the two independent cohorts mentioned above (n=37) or were additionally recruited (n=32). N= 18 were hospitalized patients with COVID-19 on normal wards (female: 39%, median age: 61), n=41 were ambulatory patients (female: 54%, median age: 36) and n=10 were SARS-CoV-2 negative controls (female: 20%, median age: 25, age of 2 subjects was unknown). The hospitalized patients were sampled at median day 11 [IQR: 6.5, 12] (hosp_sev 12.0 [IQR:11.0,13.0], hosp_normal 10.0 [IQR: 5.0, 12.0]) after symptom onset or positive RT-PCR and were hence similar to the 7-14 timepoint of ambulatory patients, which also were sampled at median day 11. The ambulatory patients were sampled at days 0-6, 7-14, as well as 60-95. The upper respiratory tract viral load of the ambulatory cohort for nasal swabs at day 0-6 was median 8.3 [IQR: 5.3, 8.9] log10(Viral load (copies/ml)), and of hospitalized patients median 5.1 [IQR: 3.8, 6.6]. A viral load time course of the ambulatory nasal swab patients is depicted in Figure 7a.

Outcomes

We did not perform a prospective outcome-based study.

Flow Cytometry

Plots

Confirm that:

- [x] The axis labels state the marker and fluorochrome used (e.g. CD4-FITC).
- [x] The axis scales are clearly visible. Include numbers along axes only for bottom left plot of group (a 'group' is an analysis of identical markers).
- [x] All plots are contour plots with outliers or pseudocolor plots.
- [x] A numerical value for number of cells or percentage (with statistics) is provided.

Methodology

Sample preparation

PBMC vials were thawed at room-temperature for 10 minutes and transferred to 5 ml PBS with 1% bovine serum albumin (BSA). Cell suspension was centrifuged at 400 x g for 15 minutes at 4 °C. Hereafter, cells were resuspended in PBS with 1% BSA and stained on ice with respective antibody master-mix panels for subsequent flow cytometry analysis and FACS-sorting.

For sorting, Cells were stained with SYTOX™ Red (Cat No. 1936399, Invitrogen) prior to sort. CD45+ living singlets were FACS/sorted and centrifuged at 400 x g for 10 minutes at 4°C. The cell concentration was adjusted to 800 cells / µl in PBS.

For murine in vivo experiments, Lung tissue was digested in RPMI 1640 containing 3.2 mg/ml Collagenase IV (Sigma) and 25 U/ml DNAse I (Sigma) for 30 minutes at 37°C. Homogenized lung was passed through 70 µm nylon mesh to obtain a single cell suspension. Cells were resuspended in 36% percoll solution (Sigma) and centrifuged for 20 minutes at 2000 rpm (light acceleration and low brake). The remaining red blood cells were removed with ACK lysis. For analysis of ex-vivo intracellular cytokine production, 1 mg/ml of brefeldin A (Sigma) were included in the digestion buffer. Cells were stimulated for 4 h at 37°C with SARS-CoV-2 PepTivator Peptide Pools (Milteny), consisting mainly of 15-mer sequences with 11 amino acids (aa) overlap, covering the immunodominant sequence domains of the surface glycoprotein "S" (#130-126-700), the membrane glycoprotein M (#130-126-702), the complete sequence of the nucleocapsid phosphoprotein N (#130-126-698), the N-terminal S1 domain of the S protein (protein S1, #130-127-041), the sequence domain aa 689–895 of the S protein (protein S+, #130-127-311) of SARS-CoV-2. Cell viability was assessed by staining with Viobility™ 405/520 fixable dye (Miltenyi).
Antibodies (Abs) used included
AF488 CD8 Biolegend #100723
PE-Cy7 CD3 Pharmingen #552774
APC/Cy7 CD44 Biolegend #103028
BV421 IFNg Biolegend #505830

Instrument	BD LSRFortessa Flow Cytometer<br>BD FACSMelody<br>BC CytoFLEX S cytom<br>BD FACSSymphony A5 SORP
Software	BD FacsDiva and Beckman CytExpert were used for aquisition
Cell population abundance	The abundance and purity of cells after sorting was as expected. Microscopic verification as well as flow cytometric verification was performed to ensure quality and quantity.
Gating strategy	Gatig strategy is outlined in the supplementary Figure 1. Cells were first gated for singlets (FSC-A vs FSC-H), then live cells were selected based on a Live/Dead stain and then leukocytes were selected according to size (FSC-A vs SSC-A). Subsequent gating for the subpopulations was done using staining for surface makers of the relevant populations.

☒ Tick this box to confirm that a figure exemplifying the gating strategy is provided in the Supplementary Information.
